# AvidinOX-anchored biotinylated trastuzumab and pertuzumab induce down-modulation of ErbB2 and tumor cell death at concentrations order of magnitude lower than not-anchored antibodies

**DOI:** 10.18632/oncotarget.15145

**Published:** 2017-02-07

**Authors:** Ferdinando Maria Milazzo, Anna Maria Anastasi, Caterina Chiapparino, Antonio Rosi, Barbara Leoni, Loredana Vesci, Fiorella Petronzelli, Rita De Santis

**Affiliations:** ^1^ Biotech Products, Research and Development, Sigma-Tau SpA, 00071 Pomezia, Rome, Italy

**Keywords:** avidinOX, trastuzumab, pertuzumab, ErbB2, cancer

## Abstract

The oxidized version of Avidin, known as AvidinOX, was previously shown to link to tissue proteins upon injection or nebulization, thus becoming a stable receptor for biotinylated therapeutics. AvidinOX is currently under clinical investigation to target radioactive biotin to inoperable tumor lesions (ClinicalTrials.gov NCT02053324). Presently, we show that the anti-ErbB2 monoclonal antibodies Trastuzumab and Pertuzumab can be chemically biotinylated while maintaining their biochemical and biological properties. By using several and diverse experimental conditions, we show that when AvidinOX is conjugated to tumor cells, low antibody concentrations of biotinylated Trastuzumab (bTrast) or Pertuzumab (bPert) prevent internalization of ErbB2, induce endoplasmic reticulum stress, cell cycle arrest and apoptosis leading to inhibition of proliferation and ErbB2 signaling. Moreover, we found that the treatment is able to induce down-modulation of ErbB2 thus bypassing the known resistance of this receptor to degradation. Interestingly, we show that AvidinOX anchorage is a way to counteract agonistic activities of Trastuzumab and Pertuzumab. Present data are in agreement with previous observations from our group indicating that the engagement of the Epidermal Growth Factor Receptor (EGFR) by AvidinOX-bound biotinylated Cetuximab or Panitumumab, leads to potent tumor inhibition both *in vitro* and in animal models. All results taken together encourage further investigation of AvidinOX-based treatments with biotinylated antibodies directed to the members of the EGFR family.

## INTRODUCTION

We previously reported that the oxidized version of Avidin, named AvidinOX, exhibits the distinctive property to form Schiff's bases with tissue proteins thus constituting a stable receptor for radiolabeled biotin [1–4]. This product is currently under investigation in phase I clinical trials for targeting ^177^Lutetium-biotinDOTA (^177^Lu-ST2210) [5] to inoperable tumor lesions and liver metastases (ClinicalTrials.gov NCT02053324). Previous data from our group also showed that AvidinOX can be employed for targeted delivery of diverse biotinylated therapeutics including cells [6] or antibodies. Particularly, several *in vitro* experiments indicated that AvidinOX-anchored anti-EGFR biotinylated antibodies like biotinylated Cetuximab (bCet) or Panitumumab (bPan), exert much higher inhibitory activity against EGFR^+^ tumor cells compared to their original version. *In vitro* results were shown to correlate with anti-tumor activity of low bCet doses, intraperitoneally injected in mice with AvidinOX-treated human larynx carcinoma xenotransplants [7]. In a severe metastatic model of lung cancer, delivery by aerosol of extremely low doses of bCet was shown to control tumor growth and significantly improve survival, when administered after nebulized AvidinOX [8].

EGFR shares structural and functional properties with other members of the receptor family (HER2/ErbB2, HER3, HER4) all having roles in cancer development and drug resistance [9, 10]. Specifically, ErbB2 is the most relevant oncogenic receptor in breast and a key player in gastric cancer [11]. A role of ErbB2 in tumor resistance has been also demonstrated in lung cancer [12–14]. ErbB2 has no known ligand and is the favored dimerization partner of the receptor family. Interestingly, while the other receptors are down-modulated upon ligand-binding, ErbB2 is resistant to down-modulation and it transfers this feature to its heterodimerization partners [15]. In the present work, we show that, consistently with previous data obtained with biotinylated anti-EGFR antibodies [7, 8], AvidinOX anchorage significantly enhances *in vitro* anti-tumor activity of biotinylated anti-ErbB2 antibodies Trastuzumab (bTrast) or Pertuzumab (bPert).

## RESULTS

### Biochemical and biological characterization of biotinylated trastuzumab (bTrast) and biotinylated pertuzumab (bPert)

**Figure 1:**
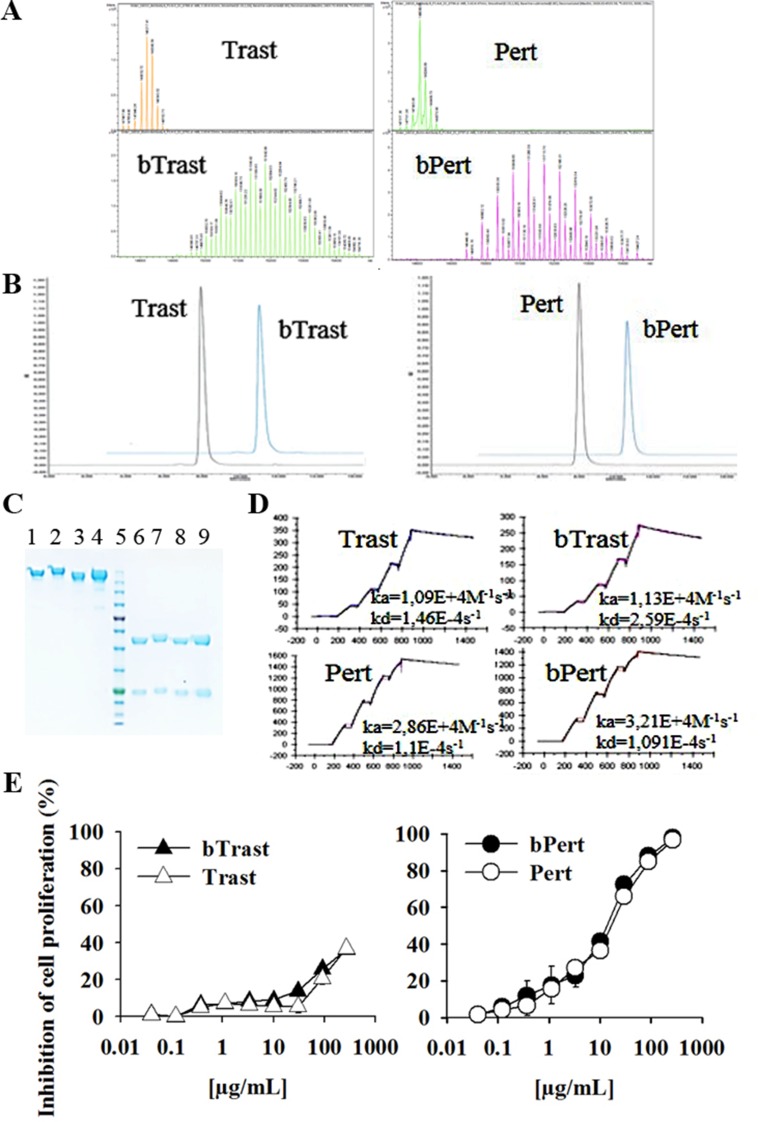
Characterization of bTrast and bPert antibodies (**A**) Electrospray Ionization Mass Spectrometry profiles of bTrast and bPert representative batches with about 8 and 7 biotins/mole, respectively, compared to Trast and Pert. (**B**) Size exclusion chromatography of bTrast and bPert representative batches as in A (blue line) compared to Trast and Pert (black line). (**C**) SDS-PAGE analysis of Trast, bTrast (lanes 1, 2), Pert, bPert (lanes 3, 4) under non-reducing conditions, and of Trast, bTrast (lanes 6, 7), Pert, bPert (lanes 8–9) in reducing conditions. Molecular weight standards in lane 5. (**D**) Analysis of HER2-ECD interaction with Trast, Pert, bTrast and bPert by SPR (Biacore). Association (ka) and dissociation (kd) constants in each sensorgram. (**E**) 32D B2/B3 cells pre-treated 2 hours with indicated antibodies and then cultivated 48 hours in the presence of 1.5 ng/mL HRG-1. Inhibition of proliferation measured by the CellTiter-Glo Luminescent Cell Viability Assay. Data are the average (± SE) of percentage inhibition of two independent experiments.

Biotinylation of Trastuzumab (Trast) and Pertuzumab (Pert) was performed as previously described for Cetuximab, Panitumumab and Rituximab [7, 8]. All batches were tested for endotoxin contamination and found to contain less than 0.008 EU/mg. Determination of the number of biotins coupled to Trastuzumab and Pertuzumab was performed by Electrospray Ionization Mass Spectrometry (ESI MS). The highest peak of Trastuzumab and Pertuzumab exhibited an estimated mass of 148217 and 148088 Da, respectively. Biotinylated forms exhibited an estimated mass of 151842 and 151260 Da with a mass difference of 3625 and 3172 Da, respectively. Since biotinylation add 452.24 Da for each added biotin, bTrast and bPert were calculated to have, in the most represented form, an average of 8.0 and 7.0 biotins/Ig molecule, respectively (Figure 1A). Size exclusion chromatography and SDS-PAGE analyses confirmed the molecular integrity of bTrast and bPert (Figure 1B and 1C, respectively). Affinity of bTrast and bPert for ErbB2 was evaluated by Surface Plasmonic Resonance (SPR, Biacore) in comparison with Trast and Pert. Antibodies were captured onto protein-A chip and their interaction with the ErbB2 extracellular domain (HER2-ECD) flowing in the cell, measured. Results in Figure 1D show similar association and dissociation kinetics to ErbB2 of original and biotinylated antibodies and lower affinity of Trast and bTrast compared to Pert and bPert.

Binding of bTrast, bPert and bCet to SKBR3 and BT474 ErbB2^+^ breast cancer cells, which express different levels of EGFR and ErbB2, was similar to the binding of the parental antibodies while, binding of the negative control anti-CD20 biotinylated Rituximab (bRit) was only visible on AvidinOX-conjugated cells, as expected (Supplementary Figure 1).

Biological activity of bTrast and bPert was tested in comparison with their respective parental antibodies by measuring proliferation of murine 32D B2/B3 transfected cells. These cells, being engineered to express human ErbB2 and ErbB3 receptors depend upon these pathways for growth [16]. Figure 1E shows similar dose response curves of original and biotinylated antibodies with IC_50_ of Pert and bPert around 15 μg/mL. Trast and bTrast were less effective inducing about 35% inhibition at 270 μg/mL. Notably, at such high concentration, the control anti-CD20 antibody Rituximab (Rit) and its biotinylated derivative (bRit) also induced about 20% proliferation inhibition (data not shown). Moreover, no anti-proliferative activity of Pert/bPert and Trast/bTrast was observed at concentrations lower than 1 and 50 μg/mL, respectively.

Finally, bTrast and bPert produced the same effects of Trast and Pert on SKBR3 signaling (i.e. phosphorylation of ErbB2, EGFR, AKT, STAT3) as evaluated by Western blotting (Supplementary Figure 2) leading to the conclusion that biotinylation does not change the biological properties of Pertuzumab or Trastuzumab.

### Inhibition of proliferation and induction of senescence, endoplasmic reticulum stress and apoptosis of ErbB2^+^ cells by AvidinOX-anchored bTrast or bPert

**Figure 2:**
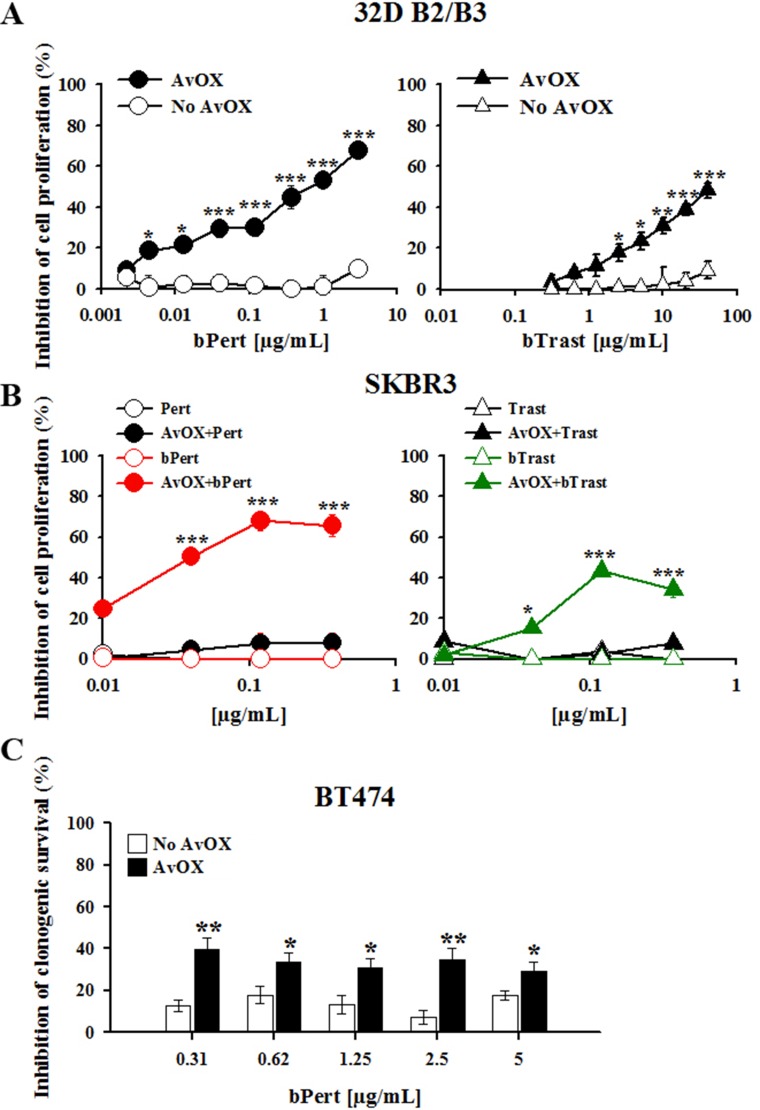
AvidinOX-anchored bTrast and bPert inhibit proliferation of ErbB2^+^ cells (**A**) 32D B2/B3 cells, with or without AvidinOX (AvOX), were cultivated 48 hours with indicated antibodies in the presence of 1.5 ng/mL HRG-1. Inhibition of proliferation measured by the CellTiter-Glo Luminescent Cell Viability Assay. Data are the average (± SE) of percentage inhibition of three independent experiments. (**B**) SKBR3 cells, with or without AvOX, were cultivated 6 days with indicated antibodies. Inhibition of proliferation measured by CellTiter-Glo Luminescent Cell Viability Assay. Data are the average (± SE) of percentage inhibition of three independent experiments. (**C**) BT474 cells (seeded at 400 cells/well, into 24-well plate) were cultivated 21 days in the presence of indicated antibodies and then clones stained with crystal violet. Optical density (595 nm) of eluted clones measured by spectrophotometer. Data are the average (± SE) of percentage inhibition (*n* = 4). Data in A–C analyzed by Mann-Whitney's test *p ≤ 0.05, **p ≤ 0.01, ***p ≤ 0.001.

To investigate the effect of AvidinOX on bTrast or bPert inhibitory activity of cell proliferation, 32D B2/B3 cells were cultivated with different antibody concentrations. Without AvidinOX, no significant inhibition was observed at concentrations up to 5 and 50 μg/mL bPert and bTrast, respectively, while, in the presence of AvidinOX, bPert induced inhibition of cell proliferation with IC_50_ at less than 1 μg/mL and bTrast induced > 40% inhibition at 40 μg/mL (Figure 2A). Under the same experimental conditions, the proliferation of wild type 32D cells, that do not express ErbB receptors, was not inhibited (data not shown). In experiments with SKBR3 cells, Trastuzumab or Pertuzumab exerted marginal inhibition of proliferation at concentrations up to 270 μg/mL, with or without AvidinOX (data not shown). Strikingly, on AvidinOX-conjugated cells, both bTrast and bPert induced significant reduction of cell proliferation at concentrations below 0.1 μg/mL (Figure 2B). Notably, similar inhibition of cell proliferation was obtained in experiments with transient exposure of AvidinOX-conjugated tumor cells to the biotinylated antibodies, performed to simulate a possible pharmacokinetic condition of patients treated intra-tumor with AvidinOX and intravenously with rapidly eliminated antibodies (Supplementary Figure 3). Additionally, the clonogenic growth of AvidinOX-conjugated BT474 cells was also significantly inhibited by bPert at concentrations as low as 0.31 μg/mL (Figure 2C).

It is to note that in AvidinOX-conjugated SKBR3 cells, the inhibition of cell proliferation was lower at higher concentrations of biotinylated antibodies. This phenomenon had been previously seen in experiments with biotinylated Cetuximab and Panitumumab and shown to be dependent on a hook effect on the AvidinOX binding [8].

**Figure 3:**
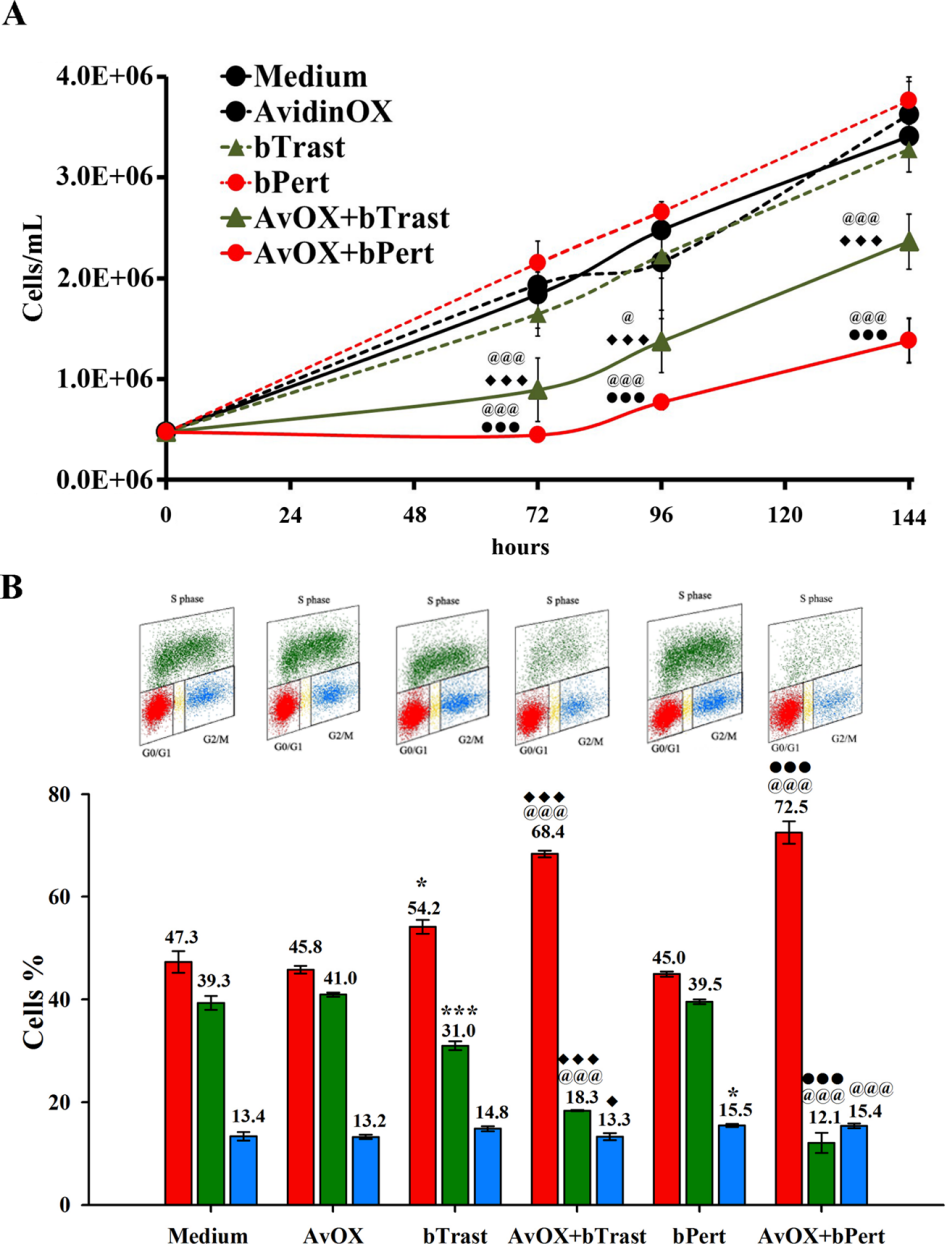
AvidinOX-anchored bTrast and bPert induce arrest of SKBR3 cell cycle (**A**) Cells, with or without AvidinOX (AvOX), were counted by nucleocounter at indicated time points after exposure to bTrast (1 μg/mL) or bPert (0.5 μg/mL). Data are the average ± SD (*n* = 3) of counted cells. (**B**) Representative flow cytometry dot plots of cell cycle phases of cells as in A, after 72 hour cultivation and staining with BrdU/PI (upper panel), and bar graph of the average ± SD (*n* = 3) of the percentage of cells in G0/G1, S and G2/M phases (lower panel). Student's *t* test: ***, *p* ≤ 0.001 and *p ≤ 0.05 vs medium; @@@, *p* ≤ 0.001 and @, *p* ≤ 0.05 vs AvidinOX; ♦♦♦, *p* ≤ 0.001 and ♦, *p* ≤ 0.05 vs bTrast; ● ● ●, *p* ≤ 0.001 vs bPert.

**Figure 4:**
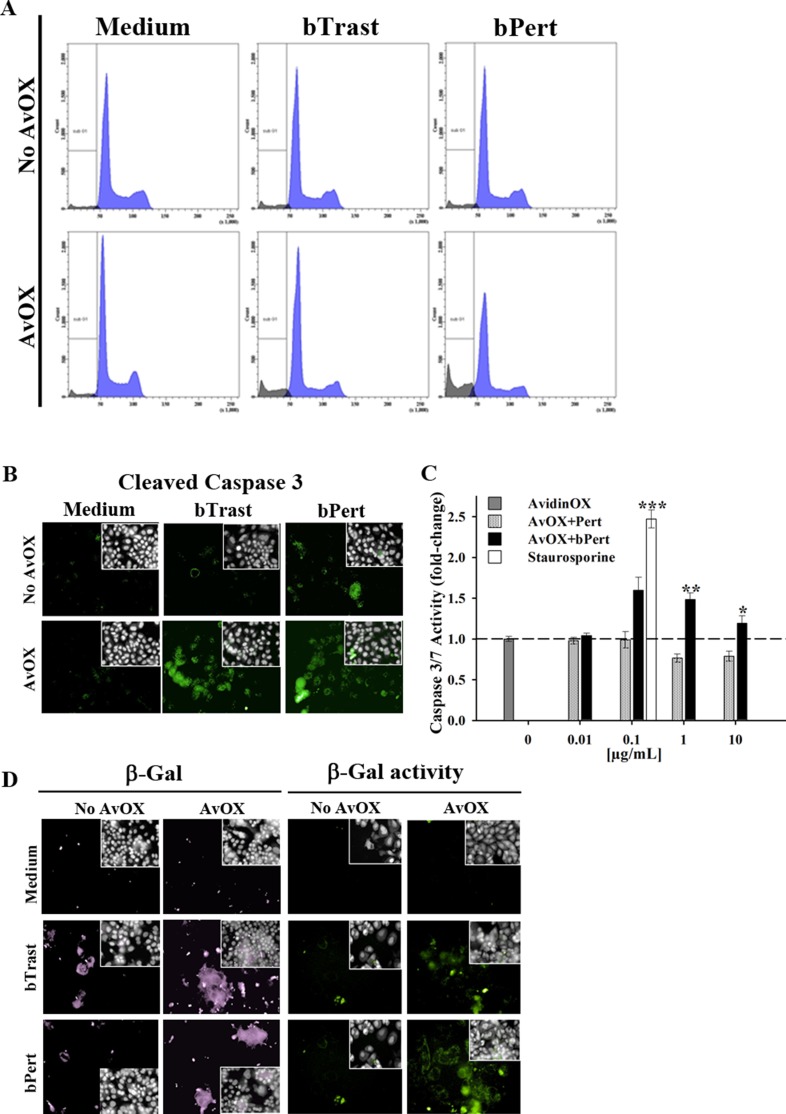
AvidinOX-anchored bTrast and bPert induce apoptosis and senescence of SKBR3 cells (**A**) Cells, with or without AvidinOX (AvOX), were stained with BrdU after 72-hour exposure to bTrast (1 μg/mL) or bPert (0.5 μg/mL) and then analyzed by flow cytometry. The Sub-G1 fraction of total cell population in grey. Representative results of one out of three independent samples. (**B**) HCS fluorescence imaging of cells cultivated 48 hours with bTrast (1 μg/mL) or bPert (0.5 μg/mL) then washed, fixed and stained with rabbit anti-cleaved caspase 3, followed by phycoherythrin-conjugated goat anti-Rabbit IgG (green). Draq5 dye staining of nucleus and cytoplasm (grey). Each image is representative of at least 5 fields of duplicate wells. Magnification 60x. Data are from one representative experiment out of two. (**C**) Caspase 3/7 activity in cells treated 72 hours with Pert or bPert at indicated concentrations, measured by Caspase-Glo 3/7 Assay. Data are expressed as fold change of activity compared to control cells and are the average of four replicates (± SE). Staurosporine (0.1 μg/mL) was included as positive control. Mann-Whitney's test: ***p ≤ 0.001, **p ≤ 0.01 and *p ≤ 0.05 vs AvOX. (**D**) Cells were cultivated 6 days with antibodies as in B, and additional 3 days without antibodies. Cells in left panel were stained with rabbit anti-β-Galactosidase antibody followed by FITC-conjugated goat anti-rabbit IgG (violet). Cells in right panel were treated 1 hour with bafilomycin A1 and additional 2 hours with C_12_FDG substrate (green). Fluorescence imaging data analysis and staining of nucleus and cytoplasm as described above. Each image is representative of at least 5 fields of duplicate wells. Magnification 60×.

Significant inhibition of AvidinOX-conjugated SKBR3 cell growth by bTrast or bPert was confirmed by cell counting (Figure 3A). Cell cycle analysis of BrdU-stained cells showed increased number of cells blocked in phase G0/G1 with a consequent dramatic reduction of the cell fraction in S phase (Figure 3B, upper and lower panels). Consistently, the percentage of apoptotic cells (PI-stained) was significantly higher compared to controls (Figure 4A), paralleled by increased levels of cleaved caspase 3/7 as found by High Content Screening (HCS) fluorescence imaging and ELISA (Figure 4B and 4C, respectively and Supplementary Figure 4). Increased apoptosis matched increased expression of the senescence marker beta-galactosidase (β-Gal) measured by HCS fluorescence imaging both as β-Gal protein and enzymatic activity (Figure 4D).

**Figure 5:**
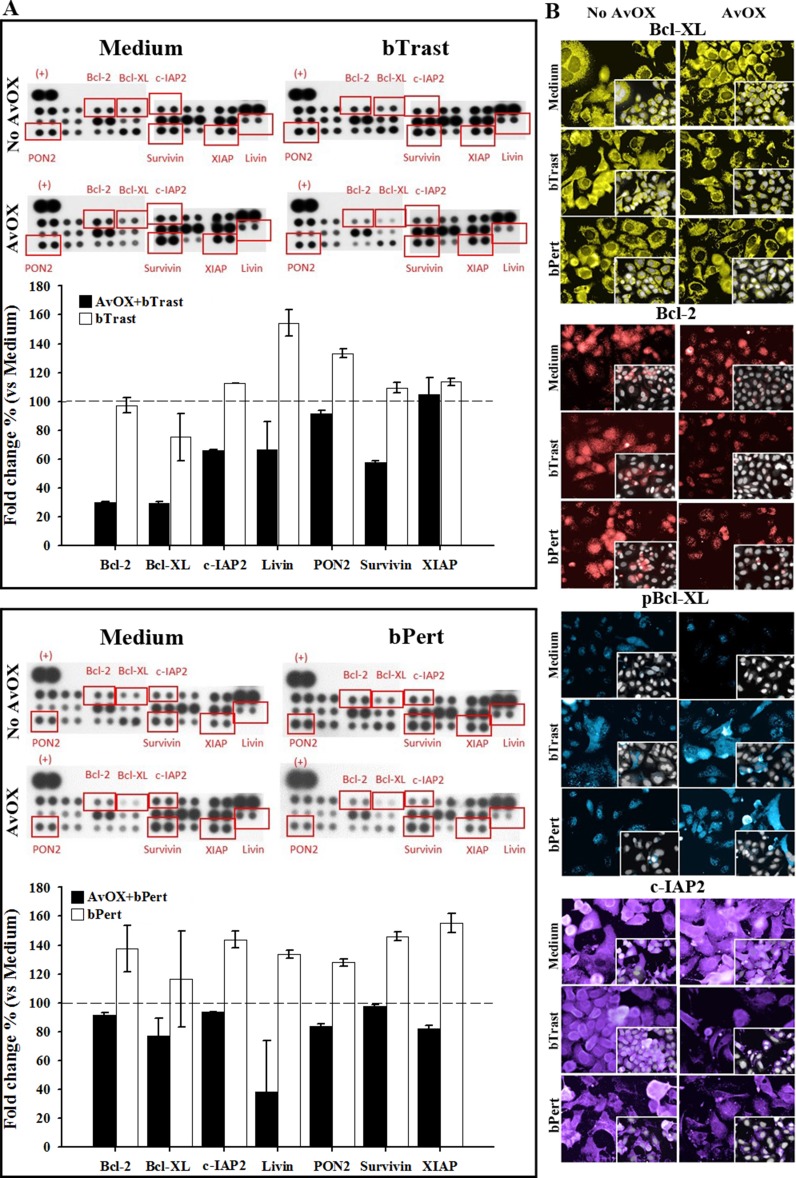
AvidinOX-anchored bTrast and bPert induce pro-apoptotic and reduce anti-apoptotic molecules in SKBR3 cells (**A**) Cells, with and without AvidinOX (AvOX) conjugation, were treated 72 hours with 1 μg/mL bTrast (upper panel) or 0.5 μg/mL bPert (lower panel), or with medium (Control). Expression of apoptosis-related proteins was measured on whole cell lysates by means of the Human Apoptosis Array kit. Representative images of the array membranes are shown. Spot pixel densities were recorded and analyzed by using image analysis software. Average background signal was subtracted from each spot and the normalized mean pixel densities of selected target proteins were plotted. Data (mean of duplicates ± SD) of selected target proteins were expressed as fold change with respect to baseline values (cells with or without AvidinOX). (**B**) Cells, with or without AvOX, were cultivated in the presence of bTrast (1 μg/mL) or bPert (0.5 μg/mL) for 72 hours and then fixed and stained with rabbit anti-Bcl-XL (yellow), rabbit anti-phospho-Bcl-XL (blue), rabbit anti-Bcl-2 (red) or mouse anti-cIAP2 (violet) followed by FITC-conjugated goat anti-rabbit IgG or goat anti-mouse IgG. Draq5 dye staining of nucleus and cytoplasm (grey). Fluorescence imaging by HCS Operetta. Each image is representative of at least 5 fields of duplicate wells. Magnification 60×. One representative experiment out of three.

Further investigation of SKBR3 apoptosis by protein microarray analyses showed that bPert and, at a lower extent, bTrast induced several anti-apoptotic proteins including members of the Bcl2 family (Bcl2 and BclXL), of the IAPs family (XIAP, cIAP2, survivin, livin) or paraoxonase-2 (PON2). In the presence of AvidinOX, such effect was counteracted and most of the proteins were below the control (Figure 5A). HCS fluorescence imaging of AvidinOX-conjugated cells confirmed the reduction of anti-apoptotic BclXL, Bcl2 and cIAP2 and increase of the pro-apoptotic phosphorylated BclXL form (pBclXL), in the presence of bTrast or bPert, compared to cells without AvidinOX (Figure 5B). Interestingly, increased cleaved caspase-3 and pBclXL were also found by HCS imaging in the low ErbB2 expressing MCF7 cells when treated with AvidinOX and either bTrast or bPert (Supplementary Figure 5). Overall data in ErbB2^+^ tumor cells point to the induction of apoptosis and inhibition of apoptosis resistance by AvidinOX-anchored bTrast or bPert.

The effect of AvidinOX-anchored anti-ErbB2 antibodies on Endoplasmic Reticulum (ER) stress was then investigated by HCS fluorescence imaging. Increased amount of phosphorylated Protein kinase-like Endoplasmic Reticulum Kinase (pPERK), Activating Transcription Factor 4 (ATF4) and Activating Transcription Factor 6α (ATF6α), all markers of ER stress leading to apoptosis, was observed (Supplementary Figures 6, 7 and 8).

### AvidinOX anchorage endows bTrast and bPert with the property to induce ErbB2 down-modulation

**Figure 6:**
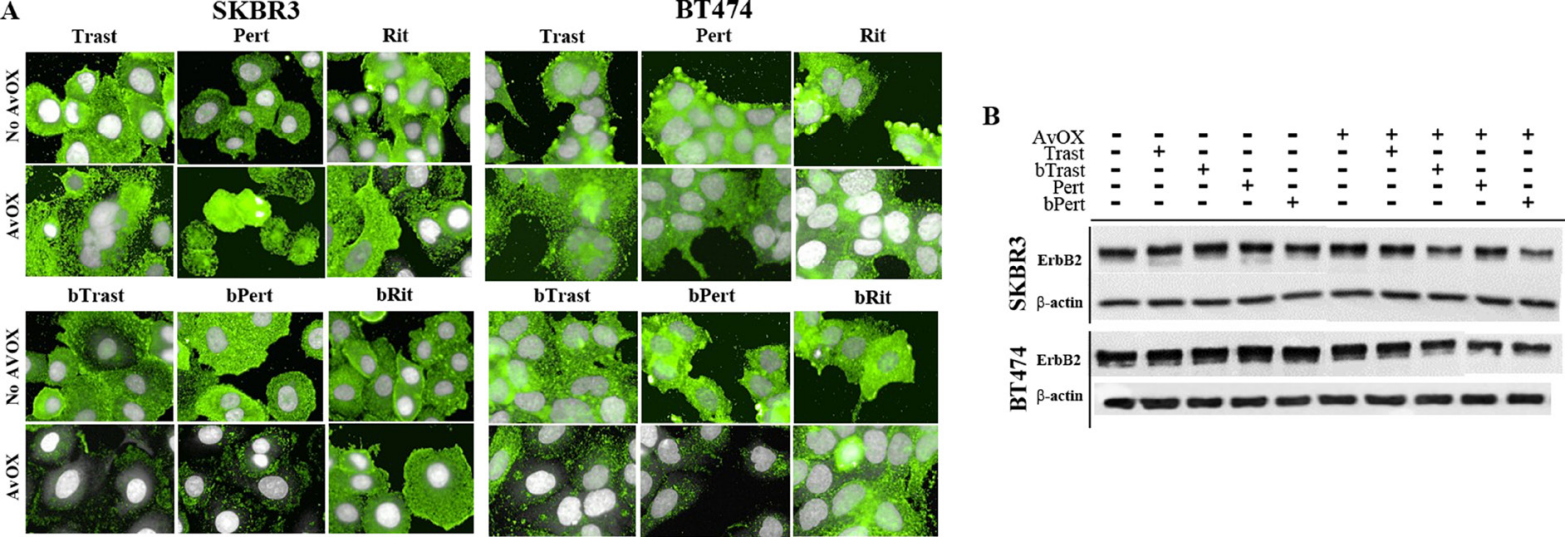
bTrast and bPert but not Trastuzumab or Pertuzumab induce degradation of ErbB2 in AvidinOX-treated cells (**A**) SKBR3 and BT474 cells, with or without AvidinOX (AvOX), were incubated 4 hours with indicated antibodies (0.2 μg/mL). After washing, cells were fixed and stained with PE-conjugated mouse anti-ErbB2 antibody (green). Draq5 dye staining of nucleus and cytoplasm (grey). Fluorescence imaging by High Content Screening (HCS) Operetta. Each image is representative of at least 5 fields of duplicate wells. Magnification 60×. Cells without antibodies (not shown) appeared similar to bRit or Rit negative control pictures. One representative experiment out of three. (**B**) SKBR3 and BT474 cells, with or without AvidinOX, were treated as in A, and then whole cell lysates were subjected to Western blot analysis with anti-ErbB2 and anti-β-actin antibodies.

**Figure 7:**
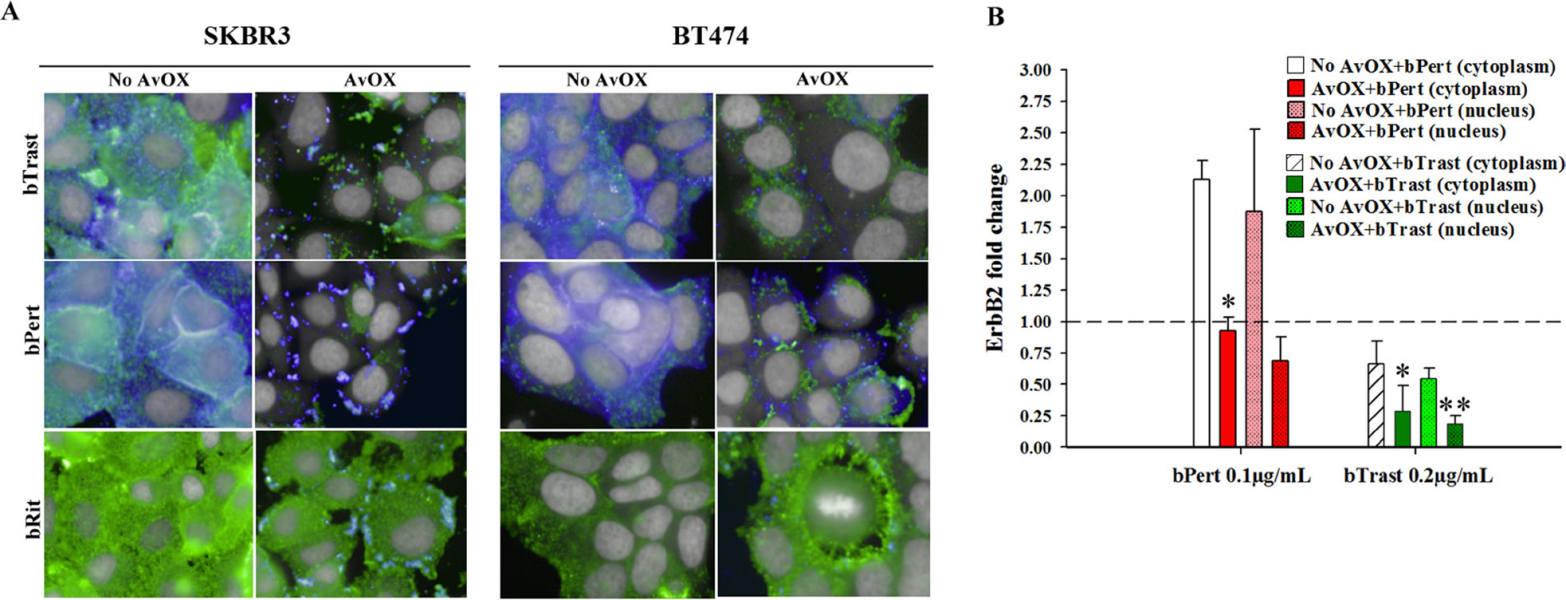
ErbB2 down modulation occurs when bTrast or bPert internalization is prevented by AvidinOX (**A**) SKBR3 and BT474 cells, with or without AvidinOX (AvOX), were incubated 30 minutes with 1 μg/mL CF488-labelled bTrast, bPert or bRit (blue) and, after washing, 4 hours with medium. Cells were then washed, fixed and stained with PE-conjugated mouse anti-ErbB2 antibody (green). Draq5 dye staining of nucleus and cytoplasm (grey). Pinkish color is the result of blue and green merge and it denotes antibody and receptor co-localization. Fluorescence imaging by HCS Operetta. Each image is representative of at least 5 fields of duplicate wells. Magnification 60x. Cells in culture medium with and without AvidinOX (not shown), were similar to bRit pictures without AvidinOX. One representative experiment out of three. (**B**) ELISA titration of ErbB2 in nuclear and cytoplasm fractions of SKBR3 cell lysates after 24-hour treatment with bTrast (0.2 μg/mL) or bPert (0.1 μg/mL) with and without AvidinOX. Data are expressed as fold change of ErbB2 compared to controls (no antibody) and are the average (± SE) of two independent experiments. Mann-Whitney's test: **p ≤ 0.01 and *p ≤ 0.05 vs No AvidinOX.

ErbB2 is known to be resistant to down-modulation [15]. Consistently, we observed by HCS fluorescence imaging, that 4-hour incubation with Trast, Pert, bTrast or bPert, did not induce down-modulation of the receptor in SKBR3 or BT474 breast cancer cells even at concentrations as high as 200 μg/mL (Supplementary Figures 9 and 10). To rule out a possible interference of AvidinOX conjugation with mechanisms of ErbB2 down-modulation, SKBR3 and BT474 cells were incubated with a HSP90 inhibitor (HSP90i) known to induce receptor degradation. Data in Supplementary Figure 11 show that HSP90i-induced ErbB2 degradation in SKBR3 cells is not affected by AvidinOX (same data in BT474, not shown). On the other hand, upon AvidinOX conjugation, 0.2 μg/mL bTrast or bPert but not Trast, Pert, Rit or bRit induced a dramatic reduction of ErbB2 in both SKBR3 and BT474 cells (Figure 6A) and similar results, after 24 hours of cell cultivation, indicated that the event is irreversible (data not shown). In agreement with HCS fluorescence imaging data, Western blot analysis showed reduction of ErbB2 in AvidinOX-conjugated SKBR3 and BT474 cells (Figure 6B). HCS fluorescence imaging showed prompt and substantial internalization of bTrast and bPert but not bRit in SKBR3 and BT474 cells (Figure 7A) and these images were comparable with those obtained with Trastuzumab and Pertuzumab (not shown). In agreement with the literature, antibody internalization did not produce significant down-modulation of ErbB2 whereas, when SKBR3 or BT474 cells were pre-treated with AvidinOX, antibody internalization was prevented and ErbB2 was dramatically down-modulated, after only 30 minutes, both in the absence (Figure 7A) or in the presence (Supplementary Figure 12) of heregulin stimulation. ELISA of SKBR3 cell lysates confirmed increased reduction of ErbB2 in both nuclear and non-nuclear compartments upon 24-hour incubation of AvidinOX-conjugated cells with bTrast or bPert (Figure 7B).

### AvidinOX anchorage of bTrast or bPert inhibits ErbB2 signaling and down-modulates pro-tumorigenic factors

**Figure 8:**
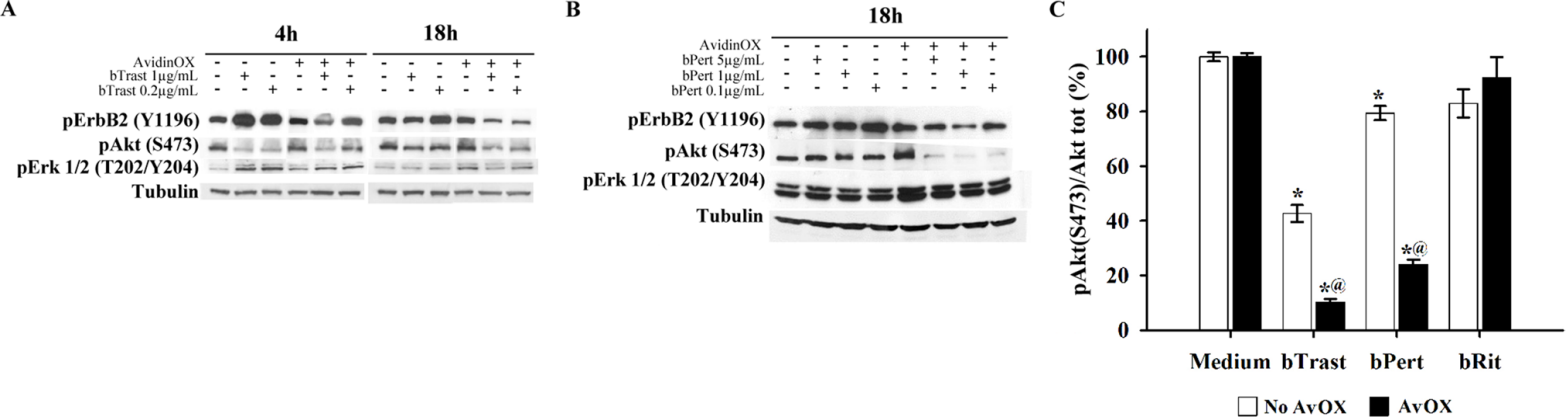
AvidinOX-anchored bTrast or bPert inhibit ErbB2 phosphorylation and signaling in SKBR3 cells Cells, with or without AvidinOX (AvOX), were incubated 1 hour with bTrast (**A**) or bPert (**B**) at the indicated antibody concentrations and then, after washing, cultivated 4–18 hours in serum-free medium. Whole cell lysates were subjected to Western blot analysis. Membranes were incubated with rabbit anti-pErbB2 (Y1196), -pAKT (S473) or -pERK1/2 (T202-Y204) antibodies followed by peroxidase-conjugated goat anti-rabbit IgG, and then revealed by ECL. Alfa tubulin was used for normalization. (**C**) Cells, with and without AvidinOX, were treated 24 hours with indicated antibodies. Total and phosphorylated Akt levels measured by ELISA (PathScan Phospho Akt (Ser473) and PathScan Total Akt kits). Data are expressed as percentage of pAkt/total Akt ratio compared to medium-treated cells, and represent the average (± SE) of two independent experiments. Mann-Whitney's test: *p ≤ 0.05 vs medium; @, *p* ≤ 0.05 vs No AvidinOX.

Western blot analysis of SKBR3 cell lysates indicated a dose-dependent inhibitory activity of ErbB2, Akt and Erk 1/2 protein phosphorylation in the presence of AvidinOX-anchored bTrast or bPert (Figure 8A and 8B, respectively). ELISA confirmed inhibition of Akt phosphorylation by AvidinOX-anchored bTrast and bPert but not bRit (Figure 8C).

**Figure 9:**
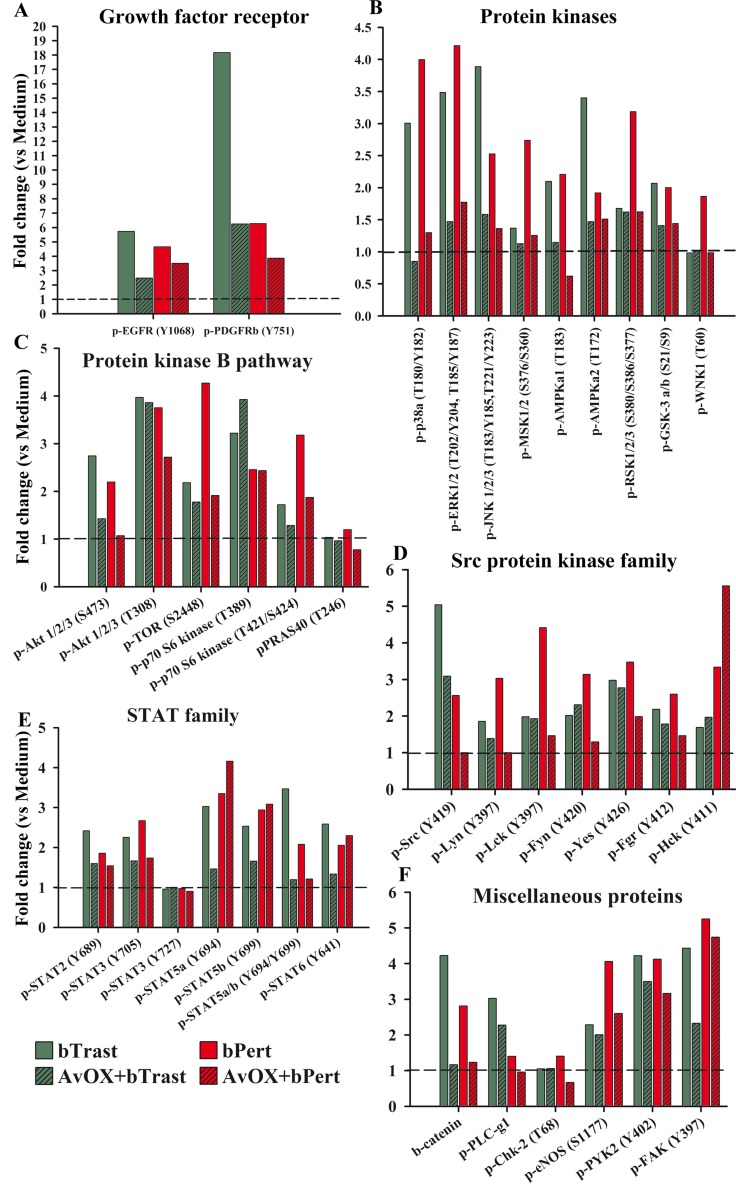
AvidinOX-anchored bTrast or bPert counteract agonistic effects of Trastuzumab or Pertuzumab on SKBR3 oncogenic pathways Cells, with and without AvidinOX (AvOX), were treated 24 hours with bTrast (1 μg/mL), bPert (0.5 μg/mL) or medium (Control). The relative levels of phosphorylation of various target proteins were measured on whole cell lysates by means of the Human Phospho-Kinase Array kit (Proteome Profiler Array). Spot pixel densities were recorded and analyzed by using image analysis software. Average background signal was subtracted from each spot and the normalized mean pixel densities were plotted according to target family. Data of selected target proteins were expressed as fold change with respect to baseline values (cells with or without AvidinOX).

To evaluate more widely the effect of AvidinOX-anchored bTrast or bPert on multiple oncogenic pathways, SKBR3 cell lysates were subjected to proteomic analyses. In the absence of AvidinOX, bTrast or bPert, similarly to Trastuzumab or Pertuzumab (as also seen in Supplementary Figure 2), induced phosphorylation of the majority of target proteins and, interestingly, such agonistic effect was largely counteracted by the presence of AvidinOX (Figure 9A–9F). Particularly, in the presence of AvidinOX, bTrast- and bPert-induced phosphorylation of EGFR (about 5 and 4 times the basal level, respectively) and Plateled Derived Growth Factor Receptor beta (PDGFRβ) (about 18 and 6 times the basal level, respectively) were clearly reduced (Figure 9A). Similar and even more striking effects were observed on several tyrosine kinase families including molecules known to be relevant to the proliferation, survival and invasion mechanisms of tumor cells like p38α, ERK1/2 and JNK1/2/3 (Figure 9B), Akt1/2/3 and TOR (Figure 9C), Src and Lyn (Figure 9D), STATs (Figure 9E) as well as βCatenin (Figure 9F). Overall, proteomic data confirmed and extended Western blot results indicating a clear counteracting effect of the agonistic activities of bTrast and bPert, upon AvidinOX anchorage.

## DISCUSSION

In the last decade, our group has been exploiting the avidin/biotin system for therapeutic approaches [1–4; 6–8; 17–24]. Currently, the oxidized version of Avidin, named AvidinOX, is being injected into inoperable liver metastases from colorectal cancer acting as a receptor for intravenously administered radioactive biotin (ClinicalTrials.gov NCT02053324). This approach represents a simplified alternative to existing local treatments relaying on the use of sophisticated medical devices (i.e. radioactive seeds) [25]. The local treatment of tumor lesions is becoming increasingly popular as part of multimodal approaches aiming at limiting systemic toxicity while promoting anti-tumor immune responses also able to act distally against untreated tumor lesions (abscopal effect) [26–31].

We previously reported anti-tumor efficacy of low biotinylated Cetuximab doses delivered by aerosol in orthotopic lung cancer models or injected intravenously in subcutaneous head and neck cancer models, after local treatment of tumors with AvidinOX [7, 8]. In the present work, we show that the monoclonal antibodies against the ErbB2/HER2 receptor Trastuzumab and Pertuzumab can be biotinylated and that when they are used in combination with AvidinOX, exhibit increased anti-tumor activity. This result could be exploited to design therapies of ErbB2^+^ tumors based on the administration of AvidinOX to the tumor lesions (i.e. intra-tumor injection of inoperable lesions or aerosol exposure of lung cancer nodules or metastases) followed by the administration (intravenous or aerosol) of an anti-ErbB2 biotinylated antibody. In a prospective use of bTrast or bPert for aerosol therapy, the molecular integrity of antibodies nebulized as phosphate buffered solutions was confirmed by SEC-HPLC analyses that also showed the formation of about 10% aggregates (Supplementary Figure 13). This result points to the need to further investigate alternative formulations as reported by other groups for this kind of products [32].

The introduction of the anti-ErbB2 antibody Trastuzumab (specific for domain IV), in combination with standard chemotherapy, significantly improved the clinical outcome of breast cancer patients. Addition of the second anti-ErbB2 antibody Pertuzumab (specific for domain II) to Trastuzumab and taxane [33] or single agent treatment with Trastuzumab-emtansine (Kadcyla) [34] further improved the median overall survival of patients with metastatic breast cancer, gaining recently regulatory approval. However, the high cost of such treatments and related pharmacoeconomy issues do not allow to foresee if these products will become part of the next standard of care [35, 36]. Therefore, strategies to reduce antibody doses like the one presently proposed should be considered.

It has been recently shown, in pre-clinical models, that the anti-tumor activity of Trastuzumab and Pertuzumab or their combination in addition to taxane can be further improved by the addition to the mixture of a third antibody, recognizing domain I [37]. Such improved efficacy correlated with induction of ErbB2 degradation, increased apoptosis and inhibition of proliferation and signaling allowing to overcome Trastuzumab resistance.

Present data show inhibition of tumor cell proliferation and signaling, induction of apoptosis and down-modulation of ErbB2 in tumor cells treated with AvidinOX and either bTrast or bPert. Interestingly, in the presence of AvidinOX, the effective concentration of each antibody is order of magnitude below the concentrations needed to see some effect, if any, without AvidinOX, with the most striking effect being the reduction of ErbB2. Down-modulation of growth factor receptors is a key mechanism for controlling the growth of tumor cells and among the receptors, ErbB2 exhibits the peculiar property to be resistant to down-modulation and to antibody-dependent ErbB2 degradation [15].

Here we show that engagement of ErbB2 by AvidinOX-anchored bTrast or bPert antibodies prevent antibody/receptor internalization (Figure 7) and induce endoplasmic reticulum stress (Supplementary Figures 6, 7 and 8) possibly leading to receptor degradation. Such possibility is supported by the observation of a pronounced reduction of ErbB2 association with the Cdc37 and HSP90 stabilizing chaperons, paralleled by increased association with HSP70 (Supplementary Figure 14) preluding to protein degradation via ubiquitin-dependent pathways [15].

Present data show for the first time that, in the presence of AvidinOX, low concentrations of a single anti-ErbB2 antibody can exhibit similar anti-tumor potency as that of high concentration antibody mixtures reported in the literature [38–40]. It is to note that AvidinOX, having four binding sites for biotin, would also allow to target simultaneously multiple receptors by using biotinylated antibodies mixtures (i.e. biotinylated Cetuximab, Trastuzumab and Pertuzumab) thus preventing or bypassing tumor resistance to a single therapeutic. Antibody Dependent Cell Cytotoxicity (ADCC) was previously shown to be not affected by the anchorage of bCet on AvidinOX [8] and we do not see reasons why bTrast or b Pert would behave differently.

In conclusion, we believe that the present work supports the rationale to use AvidinOX in combination with low dose anti-ErbB2 biotinylated antibodies for therapeutic approaches of ErbB2^+^ tumors.

## MATERIALS AND METHODS

### Biotinylated antibodies

For biotinylation of Trastuzumab (Herceptin^®^; Roche), Pertuzumab (Parjeta^®^; Roche), Cetuximab (Erbitux^®^; Merck Serono) and Rituximab (MabThera^®^; Roche) 100 mg of antibody were subjected to buffer exchange by ultrafiltration on Amicon Ultra 30K (Millipore) and brought to a concentration of about 10 mg/mL in PBS. Activated 2X-AHbiotin-N-Hydroxysuccinimide ester (ST3297, Sigma-Tau) was added at 1:10 Mab:biotin molar ratio. The reaction mixture was incubated 2 hours at room temperature, under mild shaking, loaded on SEC disposable PD-10 column (G-25 Amersham-Pharmacia) and eluted with PBS. Biotinylated antibody (bMab) fractions were pooled based on OD280 values and finally filter sterilized.

Endotoxins were tested by LAL test (Endosafe PTS/MCS Cartridges, charles river).

The biotinylation level was assessed by HABA test [41] and by Electrospray Ionization Mass Spectrometry (ESI MS). For the latter analysis, the samples were pretreated with 8M urea and then sprayed through a capillary at high voltage into the MS instrument where the mass over charge ratio (m/z) was measured. The mass of the molecule was determined using a deconvolution algorithm (MaxEnt).

Position of biotinylated lysine was analyzed by nanoLC-MS/MS peptide sequencing (Alphalyse, Denmark) and protein identification was based on a probability-scoring algorithm (www.matrixscience.com). Briefly, the protein samples were reduced and alkylated with iodoacetamide, i.e. carbamidomethylated, and subsequently digested with trypsin. The resulting peptides were first purified by reversed phase HPLC (Agilent 1200 system) using a short C8 column before being analyzed on a Q-TOF mass spectrometer (Bruker Maxis Impact system). The MS/MS spectra were used for UniProt and NCBI database searching by using the Mascot, version 2.4, software.

Purity and integrity were confirmed by SEC-HPLC. Antibodies (300 μg in 30 μL) were loaded on a TSKgel G3000 SWXL (7.8 × 300 mm) column (Tosoh Bioscience). Mobile phase of 100 mM phosphate buffer solution (pH 7.0), 300 mM NaCl/Acetonitrile (90:10), at a flow rate of 1mL/min. Detection by 280 nM absorbance. For SDS-PAGE analysis, samples were separated on a linear 4–15% acrylamide gradient gel and stained with Coomassie Brilliant Blue R250.

Specificity of biotinylated antibodies was tested by cytofluorimetry. Cell pellets, with or without AvidinOX-conjugation, were incubated 1 hour at 4°C with Mabs or bMabs. After washings, cells were incubated with mouse anti-human PE-conjugated Ig (BD). Analysis was carried out with FACSCalibur (Becton Dickinson).

Affinity was measured by Plasmon Resonance on a Biacore T200 biosensor (GE). Briefly, Trastuzumab, Pertuzumab or their biotinylated derivatives were diluted to 15 μg/mL in HBS-EP buffer and captured onto a protein A chip (GE) followed by injections of HER2 recombinant extracellular domain (Sino Biological) serially diluted from 500 nM, in single cycle kinetic modality to avoid possible interference of the regeneration procedure within the analysis. Final regenerations were performed by 10 mM glycine pH 2.5 pulses. Sensorgrams were fitted by the 1:1 Langmuir model by Biacore T200 Evaluation Software, version 1.0.

### Cells

SKBR3, BT474 and MCF7 cells were from ATCC. SKBR3 and BT474 cells were cultivated in RPMI-1640 (RPMI) 10% FBS supplemented with non- essential amino acids (Gibco) or 10 μg/mL bovine insulin (Sigma Aldrich), respectively. MCF7 cells were cultivated in DMEM 10% FBS.

32D and 32D B2/B3 cells (kindly provided by Prof Maurizio Alimandi, University of Rome La Sapienza, Italy) were routinely cultivated in RPMI 10% FBS containing 1 ng/mL interleukin-3 (IL-3, Cell Signaling). All experiments were performed starting from frozen cell stocks of working cell banks and all experiments performed on cells within 6–8 passages after thawing.

### AvidinOX conjugation to cells

AvidinOX^®^ (registered brand of Sigma Tau) was prepared by Areta International as a lyophilized form, according to previously described methods [4]. After reconstitution with water for injection, the protein was at 3.0 mg/mL, in acetate buffer pH 5.2 with mannitol and NaCl.

For AvidinOX conjugation, cells were washed with PBS or medium and incubated 1 hour at 4°C, or at 37°C, with 100 μL of 10 μg/mL AvidinOX. Cells were then washed with medium and used for further *in vitro* experiments.

### Antibody binding (FACS analysis)

Pellets of SKBR3 and BT474 cells, with or without AvidinOX conjugation, were incubated 1 hour at 4°C with Mabs or bMabs. After washings, cells were incubated with mouse anti-human PE-conjugated Ig (Becton Dickinson). Cytofluorimetry was performed with FACScalibur (Becton Dickinson).

### Proliferation, cell cycle and clonogenic assays

32D and 32D B2/B3 cells were washed with serum-free and IL3-free RPMI, starved 2 hours and then washed twice with PBS. After AvidinOX conjugation, cells in RPMI 1% FBS were seeded in 96-well plates. After 2 hour incubation with antibodies (Mabs or bMabs), 1.5 ng/mL recombinant human NRG-b1/HRG1-β (R&D Systems) were added and cells cultivated 48 hours. Growth was measured by CellTiter-Glo Luminescent Cell Viability Assay (Promega) and reading at Veritas luminometer.

SKBR3 cells, with or without AvidinOX conjugation, were cultivated 6 days with antibodies in 96 well plates, in RPMI 10% FBS. The cells were maintained in the presence of antibodies for the entire duration of the culture, or washed after initial 15 min contact. Growth was measured by CellTiter-Glo assay as before.

For cell cycle experiments, SKBR3 cells, with or without AvidinOX conjugation, were incubated up to 144 hours with Mabs or bMabs. At indicated time points, cells were stained with 30 μM bromodeoxyuridine (BrdU, Becton Dickinson) 30 minutes at 37°C, trypsinized and counted by NucleoCounter NC200 (ChemoMetec). Cell cycle and apoptosis analyses were performed by flow cytometry (FACSCanto II, Becton Dickinson) on cells fixed with 70% ethanol and stained with 2 μg/mL Propidium Iodide (PI, Sigma Aldrich) and FITC-conjugated mouse anti-BrdU antibody (Becton Dickinson).

Clonogenic growth of BT474 cells was investigated by seeding 400 cells/well (24-well plates) in RPMI 10% FBS. After 24 hours, cells with or without AvidinOX conjugation, were incubated 15 minutes with bMabs, then washed and cultivated 21 days in RPMI 10% FBS. Culture medium was then removed and cells fixed with 70% ethanol (200 μL/well, 10 minutes). Staining with 5% crystal violet in ethanol, 1 hour under mild shaking. After repeated washings with tap water, the clones were eluted with 30% acetic acid and 595 nm absorbance registered by spectrophotometer (Sunrise, Tecan).

### High content screening (HCS) fluorescence imaging

Cells were seeded in 96-well microtiter plates (0.5–1 × 10^4^/well), starved for 24 hours in serum-free medium and then, with or without AvidinOX conjugation, incubated with different concentrations of CF488-labelled or unlabelled Mabs and bMabs, in serum-free medium, for the indicated times. Cells were then fixed with 4% formaldehyde in PBS, permeabilized with PBS 0.2%Tween-20 (PBS-T) and blocked with 2% BSA in PBS-T. Unlabelled Mabs and bMabs were ultimately detected by using FITC-conjugated anti-human IgGs (BD).

Expression of protein targets after cell fixation, permeabilization and blocking as described above, was evaluated by adding the following specific primary antibodies: rabbit anti-cleaved caspase-3 (Cell Signaling), rabbit anti-β-Galactosidase (Millipore), rabbit anti-Bcl-XL (Cell Signaling), rabbit anti-p-Bcl-XL (Invitrogen), rabbit anti-Bcl-2, (abcam), mouse anti-cIAP2 (R&D Systems), rabbit anti-phospho-PERK (Santa Cruz Biot.), rabbit anti-ATF4 (proteintech), rabbit anti-ATF-6α (Santa Cruz Biot). FITC-conjugated goat anti-rabbit IgG (Becton Dickinson) or goat anti-mouse IgG (Becton Dickinson) were then added, according to the primary antibody used. EGFR and ErbB2 were detected by AF555-conjugated rabbit anti-EGFR (Cell Signaling) and PE-conjugated mouse anti-Neu (Santa Cruz Biot.), respectively. Lysosomes were stained by AF488-conjugated mouse anti-LAMP-1 (Santa Cruz Biot.).

For measuring the β-Galactosidase activity, following cultivation with antibodies as indicated, cells were treated 1 hour with bafilomycin A1 (Sigma Aldrich) and additional 2 hours with the C_12_FDG probe (Molecular Probes) that, once cleaved by the enzyme inside the cell, produces a fluorescent product substrate. Cells were counterstained with Draq5 (Cell Signaling). Fluorescence signals were acquired by the High Content Screening (HCS) system Operetta (Perkin Elmer) and images analysed through Harmony software (Perkin Elmer).

### *In situ* proximity ligation assay (PLA)

*In situ* PLA was used to evaluate protein interactions. Briefly, cells were seeded in 96-well microtiter plates (0.5–1 × 10^4^/well) and treated as described above. Cells were then washed in PBS, fixed in 4% formaldehyde in PBS, permeabilized with PBS 0.2%Tween-20 (PBS-T) and blocked with 2% BSA in PBS-T. PLA pre-qualified rabbit anti-ErbB2 antibody (Sigma Aldrich) was used together with one of the following primary antibodies, according to the protein interaction analyzed: mouse anti-Cdc37 (Santa Cruz Biot), mouse anti-HSP70 (Sigma Aldrich) or mouse anti-HSP90 (abcam). The PLA reactions were performed using Duolink^®^ In Situ reagents, from Olink^®^ Bioscience, according to manufacturer's instructions. Nuclei were stained with Duolink In Situ Microplate Nuclear Stain, Anti-Fade reagent (Sigma Aldrich). Fluorescence images were acquired by the High Content Screening (HCS) system Operetta and analysed with Harmony software.

### Western blotting

Cells were seeded in 10-cm culture plates (3 × 10^6^ cells/plate) in complete medium, and then starved 24 hours in serum-free medium. Cells, with and without AvidinOX conjugation, were then cultivated with different concentrations of antibodies, for indicated times, in serum-free medium. Cells were then washed twice with ice-cold PBS and whole cell lysates were prepared by incubation, 10 min on ice, with 1× Lysis Buffer (Cell Signaling) supplemented with protease and phosphatase inhibitors. Cell lysates were subjected to sonication prior to centrifugation at 14.000 × g, for 10 min at 4°C, to remove cell debris. Protein content was determined by Bradford method. Equal amounts of soluble proteins were separated on SDS-PAGE and then transferred to nitrocellulose membrane (Amersham Hybond-ECL; GE Healthcare). Membranes were blocked 3 hours at room temperature with 5% non-fat dry milk in PBS 0.05% Tween-20 (PBS-T) before overnight incubation, at 4°C, with one of the following primary antibodies: rabbit anti-pErbB2 (Y1196), -pAKT (S473) and –pP44/42 MAPK (Erk1/2) (T202/Y204), and mouse anti-pEGFR (Y1068) from Cell Signaling; mouse anti-ErbB2/Neu and rabbit anti-pSTAT3 (Y727) from Santa Cruz Biotechnology. Immunoblotting with rabbit anti-α-tubulin (abcam) or mouse anti-β-actin (Sigma Aldrich) was performed to normalize protein loading. After washings with PBS-T, membranes were incubated 1 hour with the appropriate secondary HRP-conjugated anti-rabbit or anti-mouse IgG antibody (Sigma Aldrich and Amersham GE-Healthcare, respectively). Immunoreactive bands were visualized by enhanced chemiluminescence detection (Amersham ECL plus; GE-Healthcare) and analyzed through phosphoimaging (STORM, Molecular Dynamics) or by exposure to X-ray film (Amersham Hyperfilm ECL; GE-Healthcare).

### Caspase 3/7 activity

For measurement of caspase 3/7 activity, SKBR3 cells (5000/well in 96-well plate) with or without AvidinOX conjugation, were cultivated 3 days in RPMI 10% FBS with antibodies and then enzymatic activity measured by Caspase-Glo 3/7 Assay (Promega), according to manufacturer's instructions. Staurosporine (Santa Cruz) was included as a positive control of apoptosis.

### ELISA titration of ErbB2 and phosphorylated-AKT

SKBR3 cells, with or without AvidinOX conjugation, were starved 24 hours and then cultivated 24 hours with antibodies or medium alone. Whole cell lysates were prepared as previously described for Western blotting analysis, and the content of phosphorylated-AKT (on Ser473 residue) and total AKT was assessed by the specific PathScan Chemiluminescent Sandwich ELISA kits (Cell Signaling), according to manufacturer's instructions.

To extract the cytoplasmic/membrane protein fraction, the cells were washed twice with PBS and resuspended in low-salt buffer (20 mM Hepes, 1 mM EDTA, 1 mM EGTA) with 0.5% Nonidet P-40, 1 mM DTT and protease and phosphatase inhibitors and were allowed to swell on ice 20 min. The cell suspension was then transferred into a syringe and slowly passed 3 times through a 28-gauge needle, and subsequently subjected to sonication 10 sec on ice, followed by centrifugation 10 min at 4°C. The supernatant [cytosol/membrane fraction] was collected and stored at −80°C. The nuclear pellet was washed 3 times with cold low-salt buffer and resuspended in high-salt lysis buffer (20 mM Hepes, 1 mM EDTA, 1 mM EGTA, 420 mM NaCl, 20% glycerol) with DTT and protease and phosphatase inhibitors. Nuclear protein extraction was achieved by passing the solution through a syringe with a 28-gauge needle, and then incubating 30 min on ice before proceeding with the final sonication and centrifugation steps as above. The supernatant was snap-frozen into aliquots and stored at −80°C. ErbB2 was then titrated in both the nuclear and non-nuclear protein fractions by using the specific PathScan Total HER2/ErbB2 chemiluminescent sandwich ELISA kit (Cell Signaling).

### Expression profiles of apoptosis-related proteins or phosphorylated kinases

SKBR3 cells were seeded in 10-cm culture plates, starved 24 hours in serum-free medium and then, with and without AvidinOX conjugation, cultivated 24–72 hours with antibodies or medium alone. Whole protein extracts were then prepared, as previously described for Western blotting analysis, and used for assessing the expression of various apoptosis-related proteins and the content of phosphorylated kinases and their substrates, through the specific Human Apoptosis and Human Phospho-Kinase Proteome Profiler Array kits (R&D Systems), respectively, according to manufacturer's instructions. After exposition of membranes to X-ray film, pixel densities were collected through the Imager 600RGB (GE) and analyzed by image analysis software (ImageQuant TL Image Analysis Software v8.1, GE). Average background signal was subtracted from each spot and the normalized mean pixel densities plotted according to target family. Data were expressed as percentage with respect to values measured in control cells.

### Statistical analysis

All data were expressed as mean values ± standard error (SE) or standard deviation (SD), and differences between groups analyzed by non-parametric Mann-Withney's test or Student's *t* test. Differences were considered significant at *p* ≤ 0.05.

### Supplementary materials

Additional data/controls on the effects of AvidinOX-anchored bTrast and bPert on tumor cells

Analytical size exclusion chromatography of nebulized bTrast and bPert

## SUPPLEMENTARY MATERIALS FIGURES



## References

[1] De Santis R, Albertoni C, Rosi A, Leoni B, Petronzelli F, D'Alessio V, Nucera E, Salvatori G, Paganelli G, Verdoliva A, Carminati P, Nuzzolo CA (2009). OXavidin for tissue targeting biotinylated therapeutics. J Biomed Biotechnol.

[2] De Santis R, Leoni B, Rosi A, Albertoni C, Forni G, Cojoca R, Iezzi M, Musiani P, Paganelli G, Chinol M, Carminati P (2010). AvidinOX for highly efficient tissue-pretargeted radionuclide therapy. Cancer Biother Radiopharm.

[3] De Santis R, Anastasi AM, Pelliccia A, Rosi A, Albertoni C, Verdoliva A, Petronzelli F, D'Alessio V, Serani S, Nuzzolo CA (2011). Chemical linkage to injected tissues is a distinctive property of oxidized avidin. PLoS One.

[4] Verdoliva A, Bellofiore P, Rivieccio V, Catello S, Colombo M, Albertoni C, Rosi A, Leoni B, Anastasi AM, De Santis R (2010). Biochemical and biological characterization of a new oxidized avidin with enhanced tissue binding properties. J Biol Chem.

[5] Urbano N, Papi S, Ginanneschi M, De Santis R, Pace S, Lindstedt R, Ferrari L, Choi S, Paganelli G, Chinol M (2007). Evaluation of a new biotin-DOTA conjugate for pretargeted antibody-guided radioimmunotherapy (PAGRIT). Eur J Nucl Med Mol Imaging.

[6] Nucera E, Nicoletti C, Chiapparino C, Pacello ML, D'Alessio V, Musarò A, De Santis R (2012). AvidinOX for tissue targeted delivery of biotinylated cells. Int J Immunopathol Pharmacol.

[7] Vesci L, Milazzo FM, Anastasi AM, Petronzelli F, Chiapparino C, Carollo V, Roscilli G, Marra E, Luberto L, Aurisicchio L, Pacello ML, Spagnoli LG, De Santis R (2016). Intra-tumor AvidinOX allows efficacy of low dose systemic biotinylated Cetuximab in a model of head and neck cancer. Oncotarget.

[8] De Santis R, Rosi A, Anastasi AM, Chiapparino C, Albertoni C, Leoni B, Pelliccia A, Santapaola D, Carollo V, Marra E, Aurisicchio L, Arseni B, Pacello ML (2014). Efficacy of aerosol therapy of lung cancer correlates with EGFR paralysis induced by AvidinOX-anchored biotinylated Cetuximab. Oncotarget.

[9] Roskoski R (2014). The ErbB/HER family of protein-tyrosine kinases and cancer. Pharmacol Res.

[10] Lemmon MA, Schlessinger J, Ferguson KM (2014). The EGFR Family: Not So Prototypical Receptor Tyrosine Kinases. Cold Spring Harb Perspect Biol.

[11] Nagaraja V, Eslick GD (2015). HER2 expression in gastric and oesophageal cancer: a meta-analytic review. J Gastrointest Oncol.

[12] Weiler D, Diebold J, Strobel K, Aebi S, Gautschi O (2015). Rapid response to trastuzumab emtansine in a patient with HER2-driven lung cancer. J Thorac Oncol.

[13] Mar N, Vredenburgh JJ, Wasser JS (2015). Targeting HER2 in the treatment of non-small cell lung cancer. Lung Cancer.

[14] Kinehara Y, Minami T, Kijima T, Hoshino S, Morimura O, Otsuka T, Hayama Y, Fukushima K, Takeuchi Y, Higashiguchi M, Miyake K, Hirata H, Nagatomo I (2015). Favorable response to trastuzumab plus irinotecan combination therapy in two patients with HER2-positive relapsed small-cell lung cancer. Lung Cancer.

[15] Bertelsen V, Stang E (2014). The Mysterious Ways of ErbB2/HER2 Trafficking. Membranes (Basel).

[16] Alimandi M, Wang LM, Bottaro D, Lee CC, Kuo A, Frankel M, Fedi P, Tang C, Lippman M, Pierce JH (1997). Epidermal growth factor and betacellulin mediate signal transduction through co-expressed ErbB2 and ErbB3 receptors. EMBO J.

[17] Albertoni C, Leoni B, Rosi A, D'Alessio V, Carollo V, Spagnoli LG, van Echteld C, De Santis R (2015). Radionuclide Therapy of Unresectable Tumors with AvidinOX and (90)Y-biotinDOTA: Tongue Cancer Paradigm. Cancer Biother Radiopharm.

[18] De Santis R, Anastasi AM, D'Alessio V, Pelliccia A, Albertoni C, Rosi A, Leoni B, Lindstedt R, Petronzelli F, Dani M, Verdoliva A, Ippolito A, Campanile N (2003). Novel antitenascin antibody with increased tumour localisation for Pretargeted Antibody-Guided RadioImmunoTherapy (PAGRIT). Br J Cancer.

[19] Paganelli G, Ferrari M, Ravasi L, Cremonesi M, De Cicco C, Galimberti V, Sivolapenko G, Luini A, De Santis R, Travaini LL, Fiorenza M, Chinol M, Papi S (2007). Intraoperative avidination for radionuclide therapy: a prospective new development to accelerate radiotherapy in breast cancer. Clin Cancer Res.

[20] Paganelli G, Ferrari M, Cremonesi M, De Cicco C, Galimberti V, Luini A, Veronesi P, Fiorenza M, Carminati P, Zanna C, Orecchia R, Veronesi U (2007). IART: intraoperative avidination for radionuclide treatment. A new way of partial breast irradiation. Breast.

[21] Paganelli G, De Cicco C, Ferrari ME, Carbone G, Pagani G, Leonardi MC, Cremonesi M, Ferrari A, Pacifici M, Di Dia A, De Santis R, Galimberti V, Luini A (2010). Intraoperative avidination for radionuclide treatment as a radiotherapy boost in breast cancer: results of a phase II study with (90)Y-labeled biotin. Eur J Nucl Med Mol Imaging.

[22] Palumbo G, Grana CM, Cocca F, De Santis R, D Del Principe, Baio SM, Mei R, Paganelli G (2007). Pretargeted antibody-guided radioimmunotherapy in a child affected by resistant anaplastic large cell lymphoma. Eur J Haematol.

[23] Petronzelli F, Pelliccia A, Anastasi AM, Lindstedt R, Manganello S, Ferrari LE, Albertoni C, Leoni B, Rosi A, D'Alessio V, Deiana K, Paganelli G, De Santis R (2010). Therapeutic Use of Avidin Is Not Hampered by Antiavidin Antibodies in Humans. Cancer Biother Radiopharm.

[24] Petronzelli F, Anastasi AM, Pelliccia A, Santapaola D, Albertoni C, Rosi A, Leoni B, Ferrari LE, Paganelli G, Gramiccioli G, Pesce D, Alfano AM, Stasi MA (2011). Preclinical Pharmacology and Safety of a Novel Avidin Derivative for Tissue-Targeted Delivery of Radiolabelled Biotin. Basic Clin Pharmacol Toxicol.

[25] Cosset JM, Flam T, Belin L, Thiounn N, Pierrat N, Pontvert D, wakil G, Savignoni A, Chauveinc L (2016). Long-term results of permanent implant prostate cancer brachytherapy: A single-institution study of 675 patients treated between 1999 and 2003. Cancer Radiother.

[26] Nakajima K, Shibamoto Y, Kobayashi M, Takaoka T, Murai T, Manabe Y, Sugie C, Yanagi T (2016). The Abscopal Effect in Patients With Multiple Metastases Treated With Combination of Dendritic Cell-Based Immunotherapy and Focal Radiation Therapy. Int J Radiat Oncol Biol Phys.

[27] Nicolay NH, Scholch S, Rauber C, Lopez PR, Debus J, Huber PE (2016). The Combination of Ionizing Radiation and Toll-Like Receptor 7/8 Agonists Creates Local and Abscopal Tumor Immune Responses In Vivo. Int J Radiat Oncol Biol Phys.

[28] Rodriguez-Ruiz ME, Rodriguez I, Garasa S, Barbes B, Solorzano JL, Perez-Gracia JL, Labiano S, Sanmamed MF, Azpilikueta A, Bolaños E, Sanchez-Paulete AR, Aznar MA, Rouzaut A (2016). Abscopal Effects of Radiotherapy Are Enhanced by Combined Immunostimulatory mAbs and Are Dependent on CD8 T Cells and Crosspriming. Cancer Res.

[29] Popp I, Grosu AL, Niedermann G, Duda DG (2016). Immune modulation by hypofractionated stereotactic radiation therapy: Therapeutic implications. Radiother Oncol.

[30] Ray A, Williams MA, Meek SM, Bowen RC, Grossmann KF, Andtbacka RH, Bowles TL, Hyngstrom JR, Leachman SA, Grossman D, Bowen GM, Holmen SL, VanBrocklin MW (2016). A phase I study of intratumoral ipilimumab and interleukin-2 in patients with advanced melanoma. Oncotarget.

[31] Johnson CB, Jagsi R (2016). The Promise of the Abscopal Effect and the Future of Trials Combining Immunotherapy and Radiation Therapy. Int J Radiat Oncol Biol Phys.

[32] Respaud R, Marchand D, Parent C, Pelat T, Thullier P, Tournamille JF, Viaud-Massuard MC, Diot P, Si-Tahar M, Vecellio L, Heuzé-Vourc'h N (2014). Effect of formulation on the stability and aerosol performance of a nebulized antibody. MAbs.

[33] Swain SM, Baselga J, Kim SB, Ro J, Semiglazov V, Campone M, Ciruelos E, Ferrero JM, Schneeweiss A, Heeson S, Clark E, Ross G, Benyunes MC (2015). Pertuzumab, trastuzumab, and docetaxel in HER2-positive metastatic breast cancer. N Engl J Med.

[34] Guerin M, Sabatier R, Goncalves A (2015). Trastuzumab emtansine (Kadcyla) approval in HER2-positive metastatic breast cancers. Bull Cancer.

[35] Fleeman N, Bagust A, Beale S, Dwan K, Dickson R, Proudlove C, Dundar Y (2015). Pertuzumab in combination with trastuzumab and docetaxel for the treatment of HER2-positive metastatic or locally recurrent unresectable breast cancer. Pharmacoeconomics.

[36] Miranda RP, Marin GR (2015). Trastuzumab emtansine in locally advanced or metastatic HER2 positive breast cancer; GENESIS-SEFH drug evaluation report. Farm Hosp.

[37] Pedersen MW, Jacobsen HJ, Koefoed K, Dahlman A, Kjaer I, Poulsen TT, Meijer PJ, Nielsen LS, Horak ID, Lantto J, Kragh M (2015). Targeting Three Distinct HER2 Domains with a Recombinant Antibody Mixture Overcomes Trastuzumab Resistance. Mol Cancer Ther.

[38] Brockhoff G, Heckel B, Schmidt-Bruecken E, Plander M, Hofstaedter F, Vollmann A, Diermeier S (2007). Differential impact of Cetuximab, Pertuzumab and Trastuzumab on BT474 and SK-BR-3 breast cancer cell proliferation. Cell Prolif.

[39] Nejatollahi F, Jaberipour M, Asgharpour M (2014). Triple blockade of HER2 by a cocktail of anti-HER2 scFv antibodies induces high antiproliferative effects in breast cancer cells. Tumour Biol.

[40] Szymanska M, Fosdahl AM, Nikolaysen F, Pedersen MW, Grandal MM, Stang E, Bertelsen V (2016). A combination of two antibodies recognizing non-overlapping epitopes of HER2 induces kinase activity-dependent internalization of HER2. J Cell Mol Med.

[41] Green NM (1965). A spectrophotometric assay for avidin and biotin based on binding of dyes by avidin. Biochem J.

