# Supplementary material for: AvidinOX-anchored biotinylated trastuzumab and pertuzumab induce down-modulation of ErbB2 and tumor cell death at concentrations order of magnitude lower than not-anchored antibodies
Source: Oncotarget. 2017 Feb 7;8(14):22590–605. doi: 10.18632/oncotarget.15145 (PMC5410247; doi:10.18632/oncotarget.15145)
Supplement: Supplementary file 1 [file oncotarget-08-22590-s001.pdf]

AvidinOX-anchored biotinylated trastuzumab and pertuzumab induce down-modulation of ErbB2 and tumor cell death at concentrations order of magnitude lower than not-anchored antibodies

Supplementary Materials

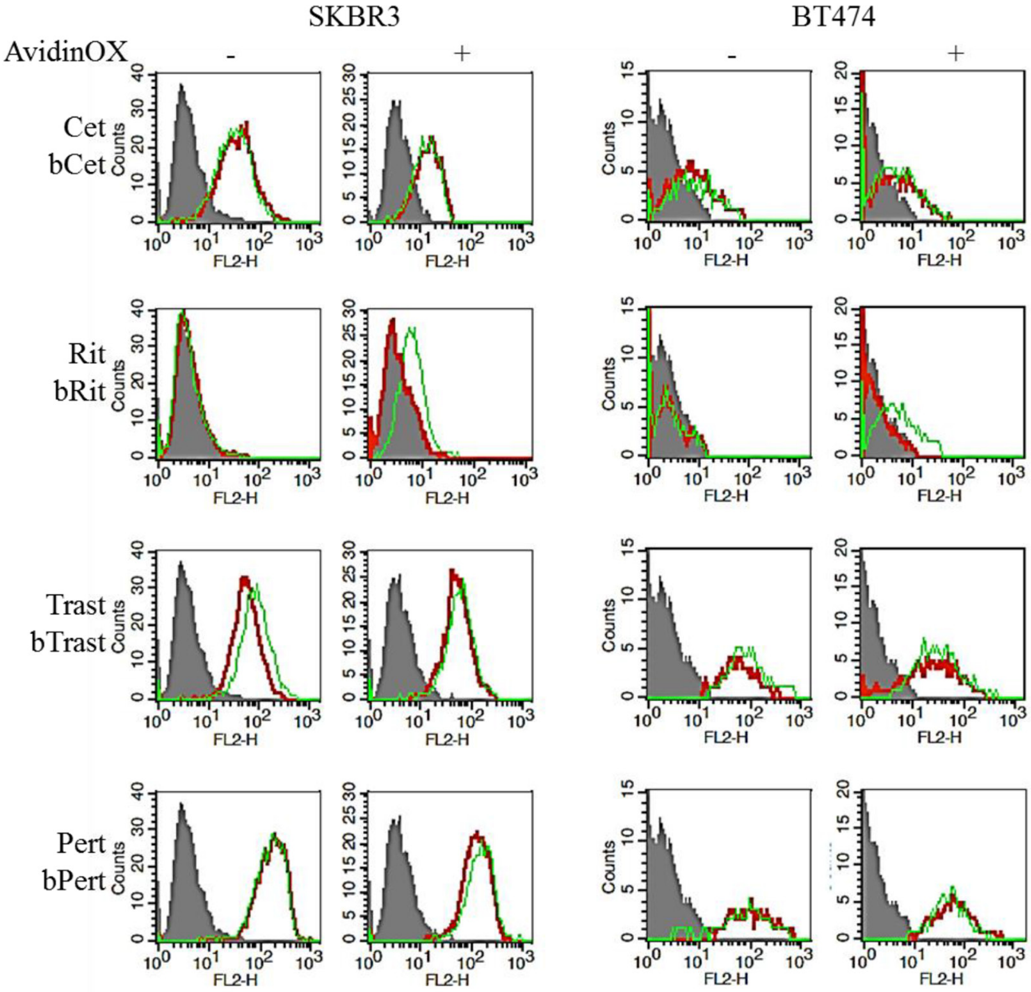

Supplementary Figure 1: Binding of biotinylated antibodies to breast cancer cells with and without AvidinOX. Flow cytometry of SKBR3 and BT474 cells incubated with native (red line) or biotinylated (green line) antibodies with and without AvidinOX conjugation. Antibody binding detected by phycoerythrin-conjugated mouse anti-human Ig (BD). Grey peaks refer to cells without primary antibody.

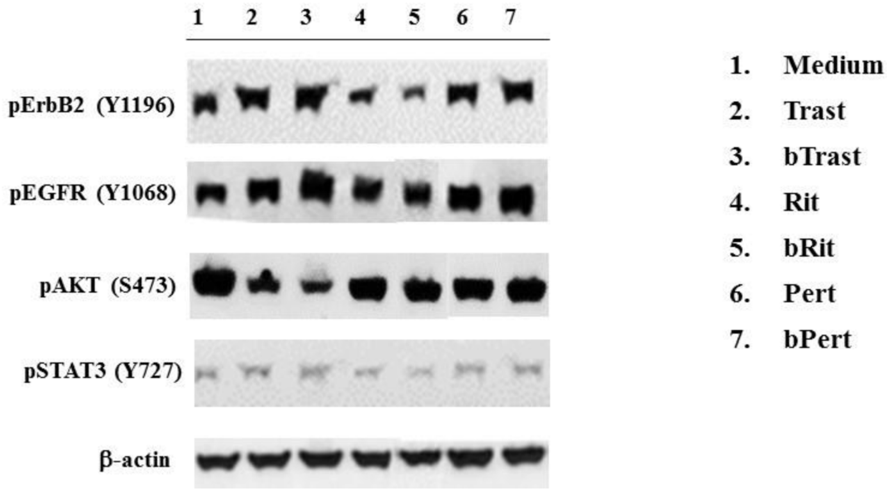

Supplementary Figure 2: Biotinylated and not-biotinylated Trastuzumab and Pertuzumab produce the same effects on SKBR3 signaling. Cells were incubated 24 hours with 1 μg/mL biotinylated or not-biotinylated Mabs in serum-free medium. Whole cell lysates were subjected to Western blot analysis. Membranes were incubated with the following antibodies: rabbit anti-pErbB2 (Y1196), -pAKT (S473) or -pSTAT3 (Y727), and mouse anti-pEGFR (Y1068). Peroxidase-conjugated goat anti-rabbit or anti-mouse IgG were then added, and immunoreactive bands revealed by ECL. Beta-actin was used for normalization.

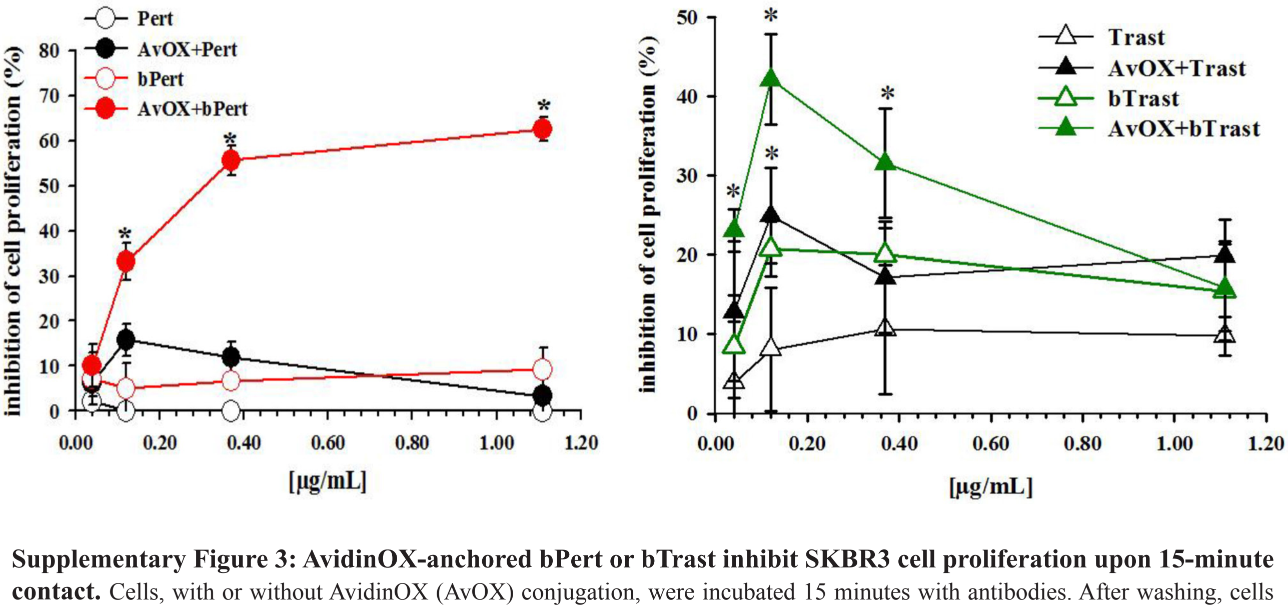

Supplementary Figure 3: AvidinOX-anchored bPert or bTrast inhibit SKBR3 cell proliferation upon 15-minute contact. Cells, with or without AvidinOX (AvOX) conjugation, were incubated 15 minutes with antibodies. After washing, cells were cultivated 6 days in culture medium and inhibition of proliferation measured by CellTiter-Glo Luminescent Cell Viability Assay. Data are the average (± SE) of percentage inhibition of two independent experiments. Mann-Whitney's test: *$p \leq 0.05$ vs No AvidinOX.

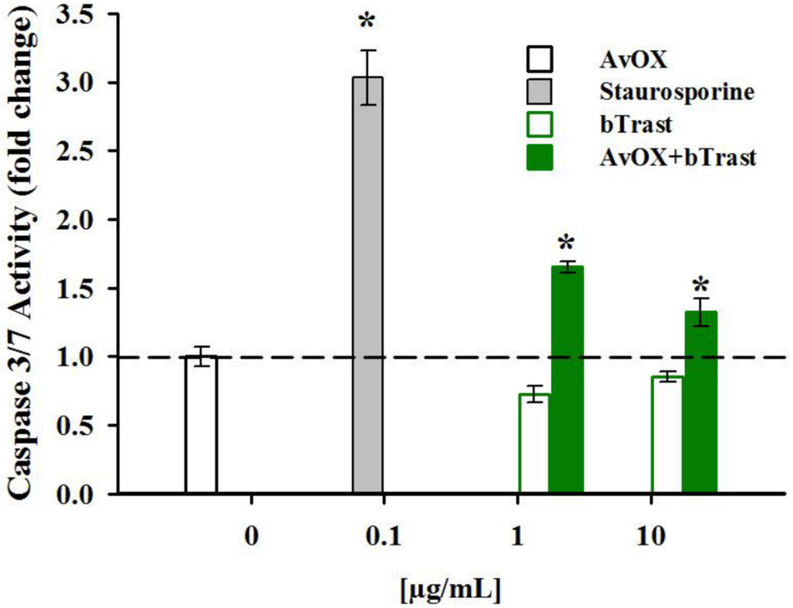

Supplementary Figure 4: AvidinOX-anchored bTrast induces caspase 3/7 activity in SKBR3 cells. Cells, with or without AvidinOX (AvOX), were cultivated 72 hours with bTrast. Caspase 3/7 activity was measured by Caspase-Glo 3/7 luminescent assay. Data are expressed as fold change of activity compared to control (AvOX) and are the average of four replicates (± SE). Staurosporine (0.1 µg/mL) was included as positive control. Mann-Whitney's test: *$p \leq 0.05$ vs AvOX.

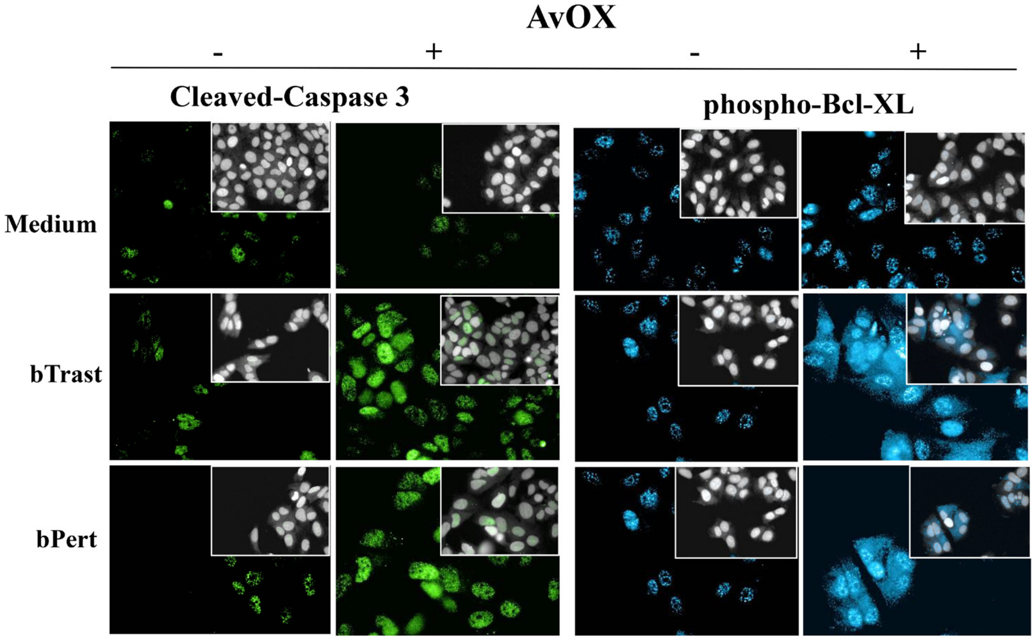

Supplementary Figure 5: AvidinOX-anchored bPert or bTrast induce apoptosis in MCF7 cells. Cells, with or without AvidinOX (AvOX), were treated 48 hours with bTrast or bPert (1µg/mL). Cells were then washed, fixed and incubated with rabbit anti-cleaved caspase 3 or anti-phospho-Bcl-XL antibodies followed by FITC-conjugated goat anti-rabbit IgG (green and blue, respectively). Draq5 dye staining of nucleus and cytoplasm (grey). Fluorescence imaging by High Content Screening (HCS) Operetta. Each image is representative of at least 5 fields of duplicate wells. Magnification 60x. Data are from one representative experiment out of two.

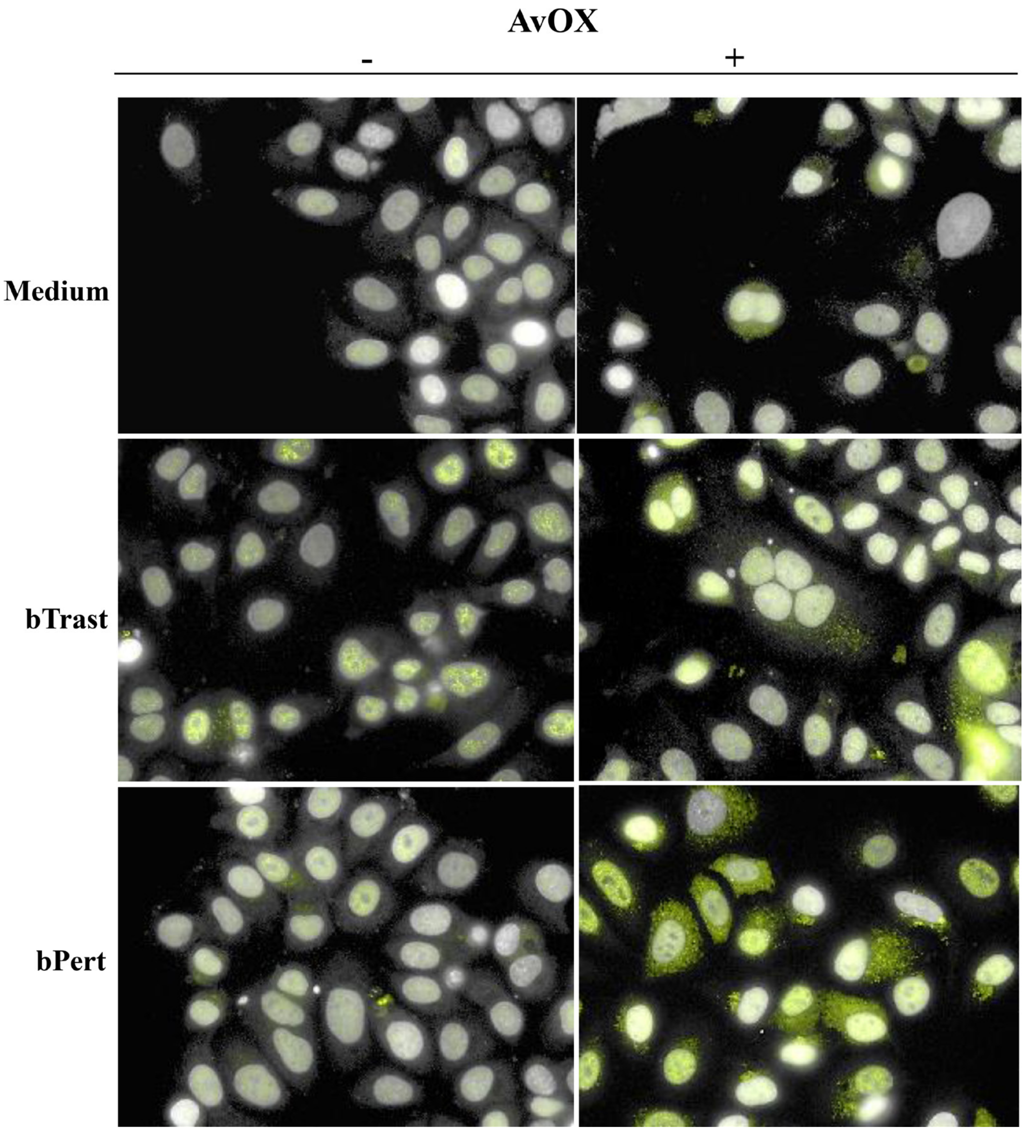

Supplementary Figure 6: AvidinOX-anchored bPert or bTrast induce pPERK in SKBR3 cells. Cells, with or without AvidinOX (AvOX) conjugation, were treated 24 hours with bTrast or bPert (1µg/mL), then washed, fixed and incubated with rabbit anti-pPERK antibody followed by FITC-conjugated goat anti-rabbit IgG (yellow). Draq5 dye staining of nucleus and cytoplasm (grey). Fluorescence imaging by High Content Screening (HCS) Operetta. Each image is representative of at least 5 fields of duplicate wells. Magnification 60x. Data are from one representative experiment out of two.

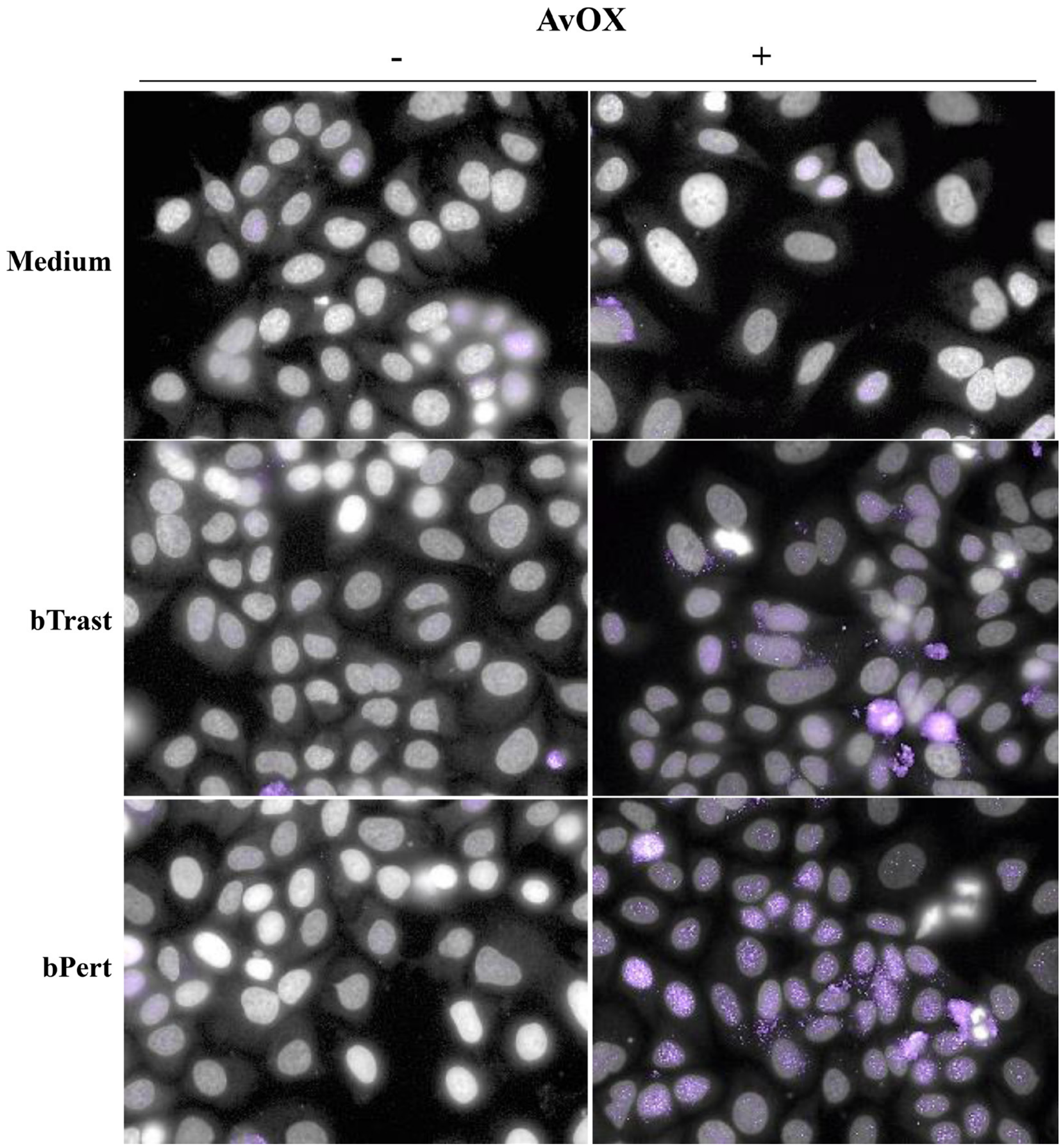

Supplementary Figure 7: AvidinOX-anchored bPert or bTrast upregulate ATF4 in SKBR3 cells. Cells, with or without AvidinOX (AvOX) conjugation, were treated 24 hours with bTrast or bPert (1µg/mL), then washed, fixed and incubated with rabbit anti-ATF4 antibody followed by FITC-conjugated goat anti-rabbit IgG (pink). Draq5 dye staining of nucleus and cytoplasm (grey). Fluorescence imaging by High Content Screening (HCS) Operetta. Each image is representative of at least 5 fields of duplicate wells. Magnification 60×. Data are from one representative experiment out of two.

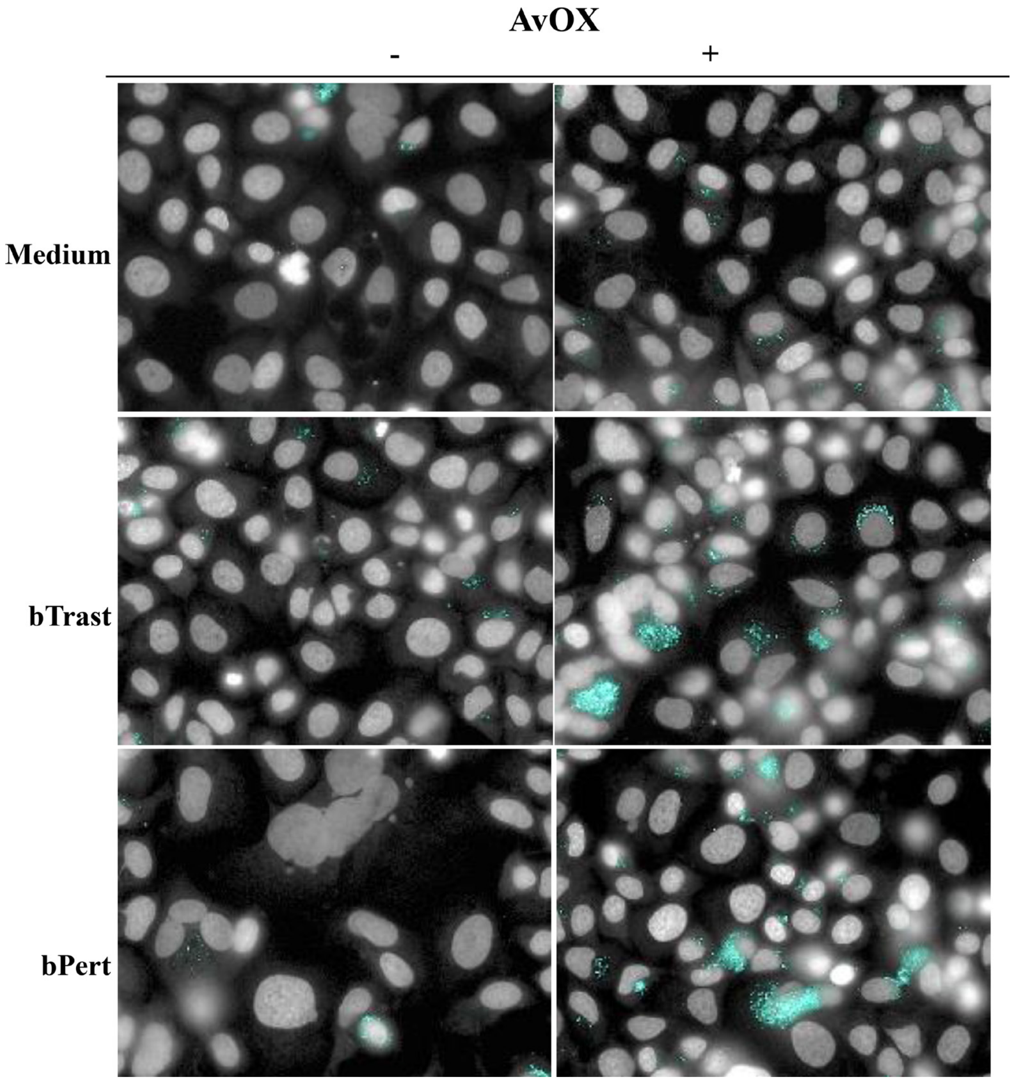

Supplementary Figure 8: AvidinOX-anchored bPert or bTrast upregulate ATF6α in SKBR3 cells. Cells, with or without AvidinOX (AvOX) conjugation, were treated 24 hours with bTrast or bPert (1µg/mL) and then cultivated additional 48 hours without antibodies. Cells were then washed, fixed and incubated with rabbit anti-ATF6α antibody followed by FITC-conjugated goat anti-rabbit IgG (light blue). Draq5 dye staining of nucleus and cytoplasm (grey). Fluorescence imaging by High Content Screening (HCS) Operetta. Each image is representative of at least 5 fields of duplicate wells. Magnification 60x. Data are from one representative experiment out of two.

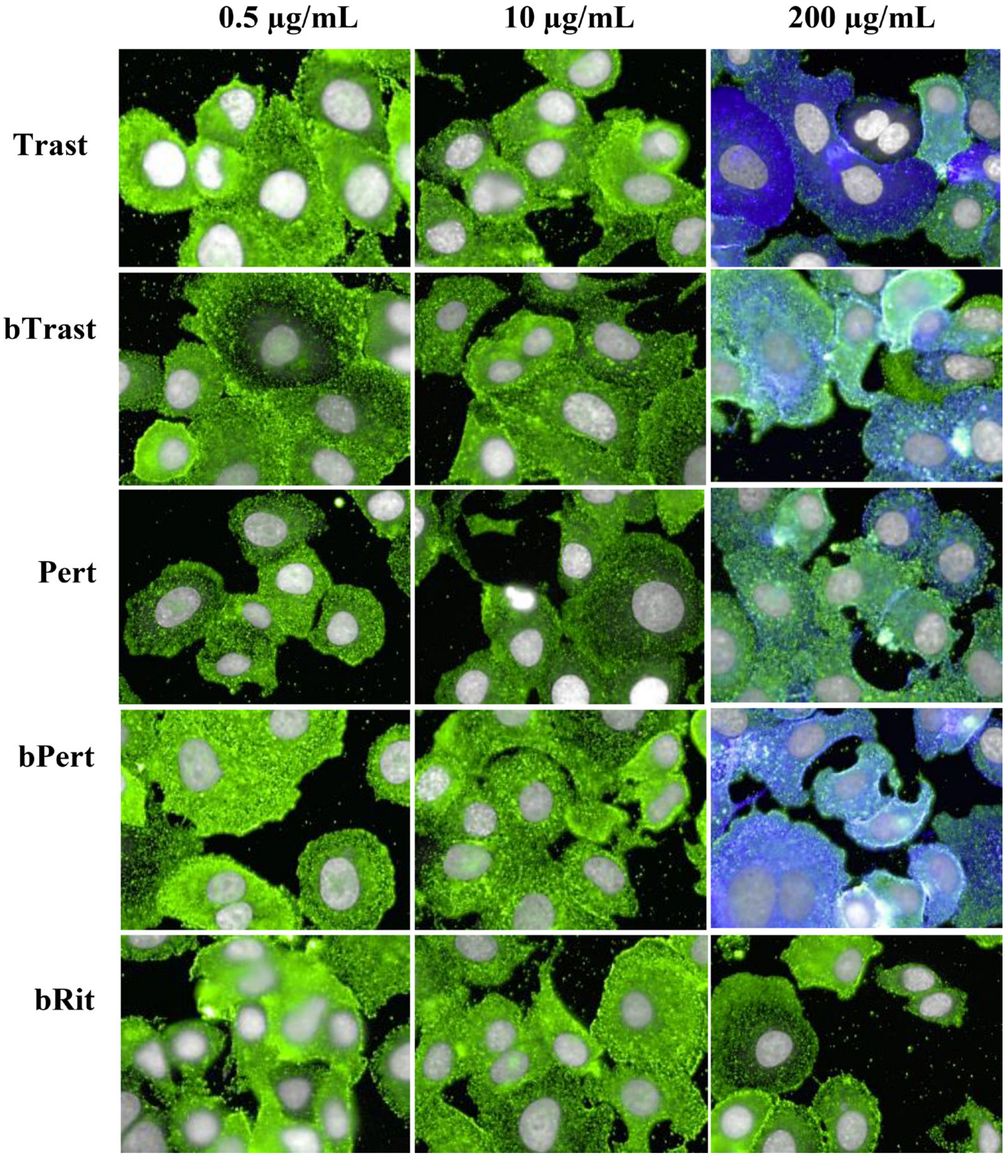

Supplementary Figure 9: ErbB2 protein is not down-modulated by anti-ErbB2 antibodies in SKBR3 cells. Cells were incubated 4 hours with indicated antibodies. After washing, cells were fixed and stained with FITC-conjugated mouse anti-human IgG (blue) for the detection of antibodies, and with PE-conjugated mouse anti-ErbB2 antibody (green). Draq5 staining of nucleus and cytoplasm (grey). Fluorescence images acquired by High Content Screening (HCS) Operetta. Each picture is representative of at least 5 fields of duplicate wells. 60× magnification. Cells without antibodies or with Rit (not shown) looked as bRit negative control. Blue staining shown at the highest concentration only, to prove antibody presence.

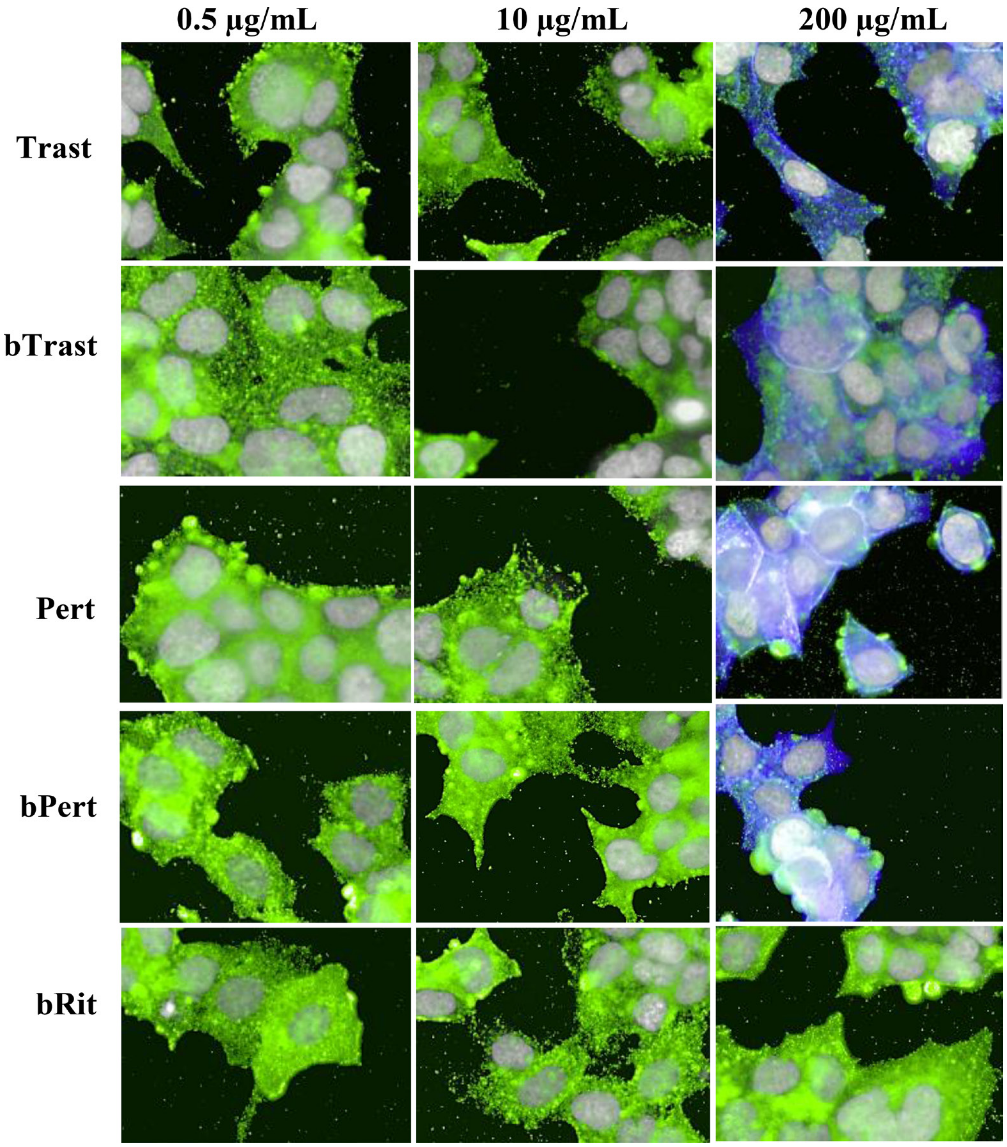

Supplementary Figure 10: ErbB2 protein is not down-modulated by anti-ErbB2 antibodies in BT474 cells. Cells were incubated 4 hours with indicated antibodies. After washing, cells were fixed and stained with FITC-conjugated mouse anti-human IgG (blue) for the detection of antibodies, and with PE-conjugated mouse anti-ErbB2 antibody (green). Draq5 staining of nucleus and cytoplasm (grey). Fluorescence images acquired by High Content Screening (HCS) Operetta. Each picture is representative of at least 5 fields of duplicate wells. 60× magnification. Cells without antibodies or with Rit (not shown) looked as bRit negative control. Blue staining shown at the highest concentration only, to prove antibody presence.

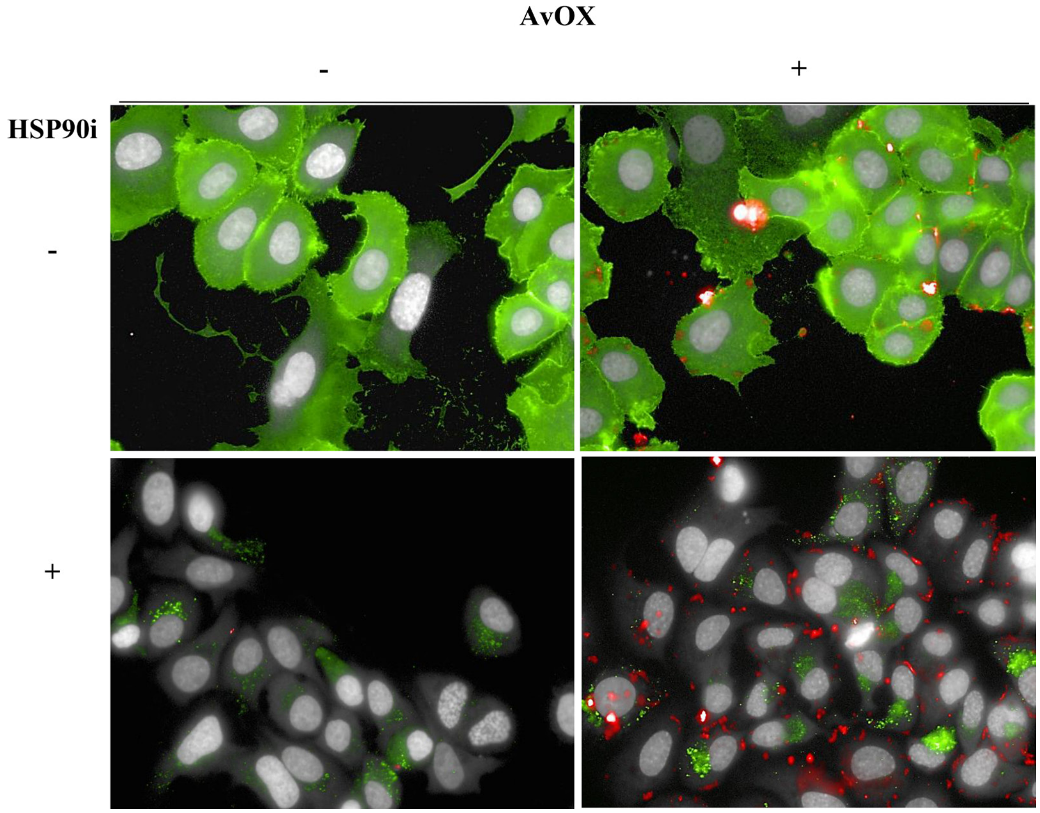

Supplementary Figure 11: ErbB2 degradation induced by HSP90i in SKBR3 cells is not affected by AvidinOX conjugation. Cells, with or without AvidinOX (AvOX) conjugation, were incubated 4 hours with HSP90i (1 μM). After washing, the cells were fixed and stained for AvidinOX by using PE-conjugated biotin (red) and for ErbB2 by using rabbit anti-ErbB2 antibody followed by FITC-conjugated goat anti-rabbit IgG (green). Draq5 staining of nucleus and cytoplasm (grey). Fluorescence images acquired by High Content Screening (HCS) Operetta. Each picture is representative of at least 5 fields of duplicate wells. 60× magnification.

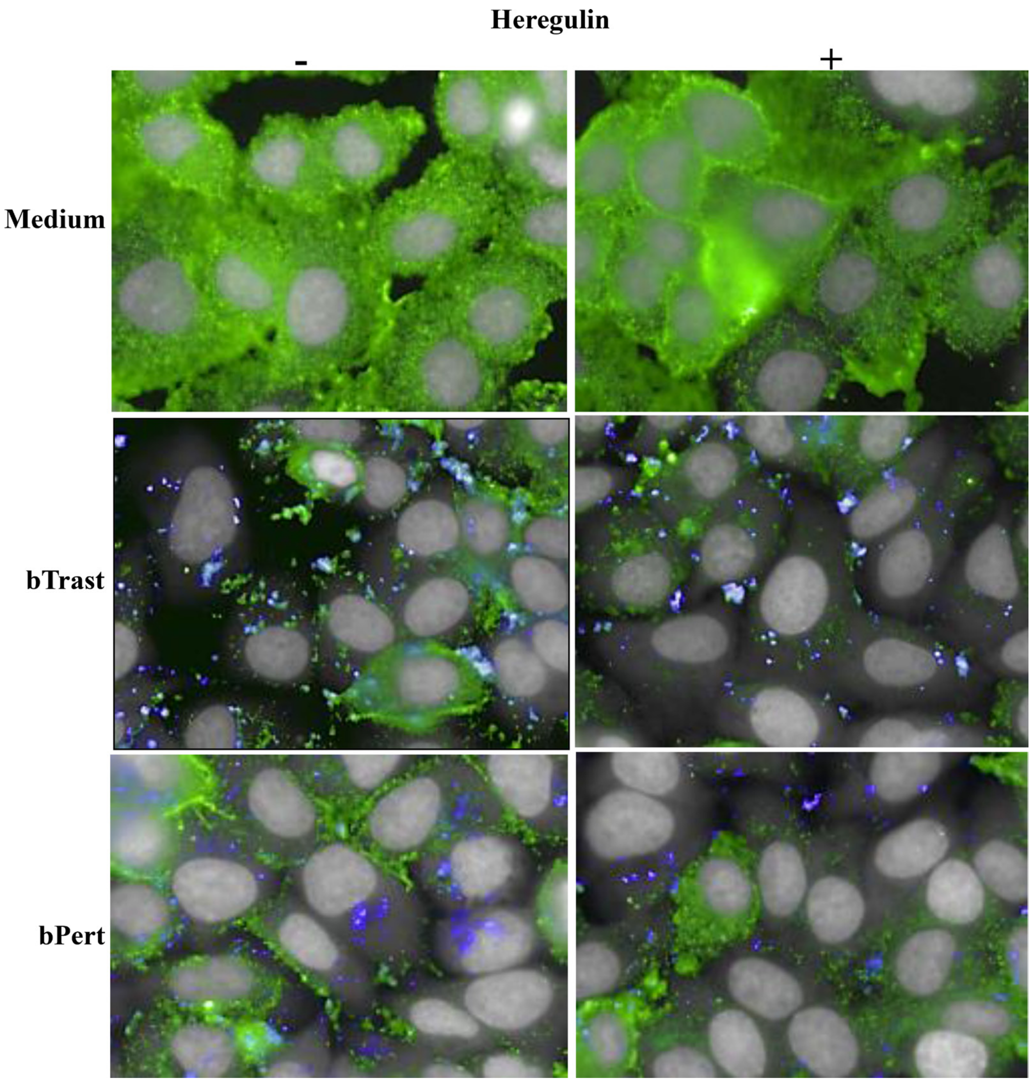

Supplementary Figure 12: ErbB2 down-modulation induced by bTrast or bPert in AvidinOX-conjugated SKBR3 cells occurs also upon Heregulin stimulation. Cells were incubated 30 minutes with 5 µg/mL CF488-labeled bTrast or bPert (blue) and, after washing, cultivated 4 hours in medium with or without Heregulin (20 ng/mL). Cells were then washed, fixed and stained for ErbB2 by using PE-conjugated mouse anti-ErbB2 antibody (green). Draq5 staining of nucleus and cytoplasm (grey). Fluorescence images acquired by HCS Operetta. Each picture is representative of at least 5 fields of duplicate wells. 60× magnification.

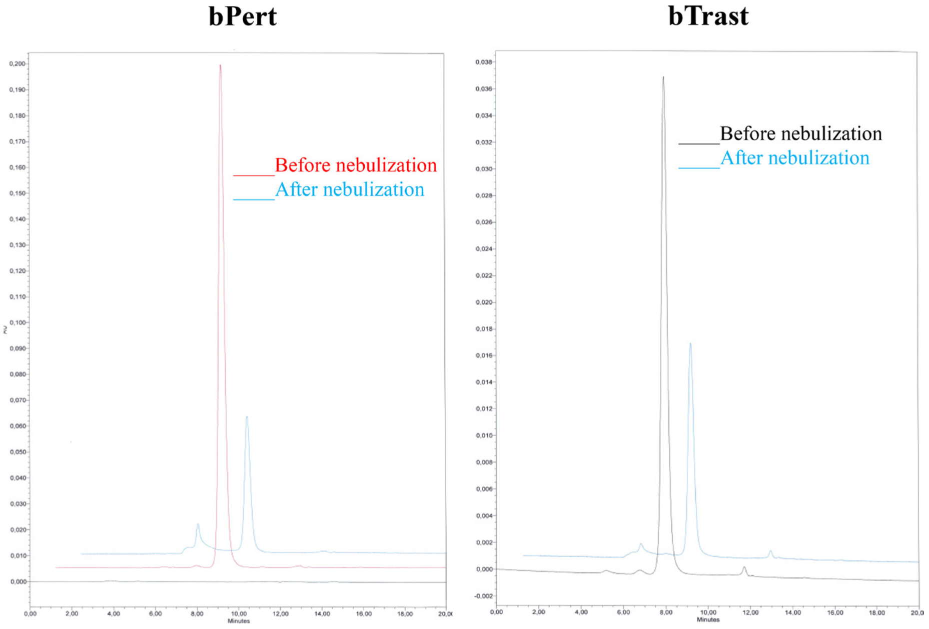

Supplementary Figure 13: Nebulized bPert and bTrast substantially maintain molecular integrity. bPert and bTrast (1 mg/mL) solutions were nebulized by using a domestic nebulizer (AirFamily system, Pic indolor). Nebulized material was collected by conveying the mist in a falcon tube and condensed solutions analyzed by SEC-HPLC (TSKgel G3000SWXL column, Tosoh Bioscience) in comparison to pre-nebulized samples.

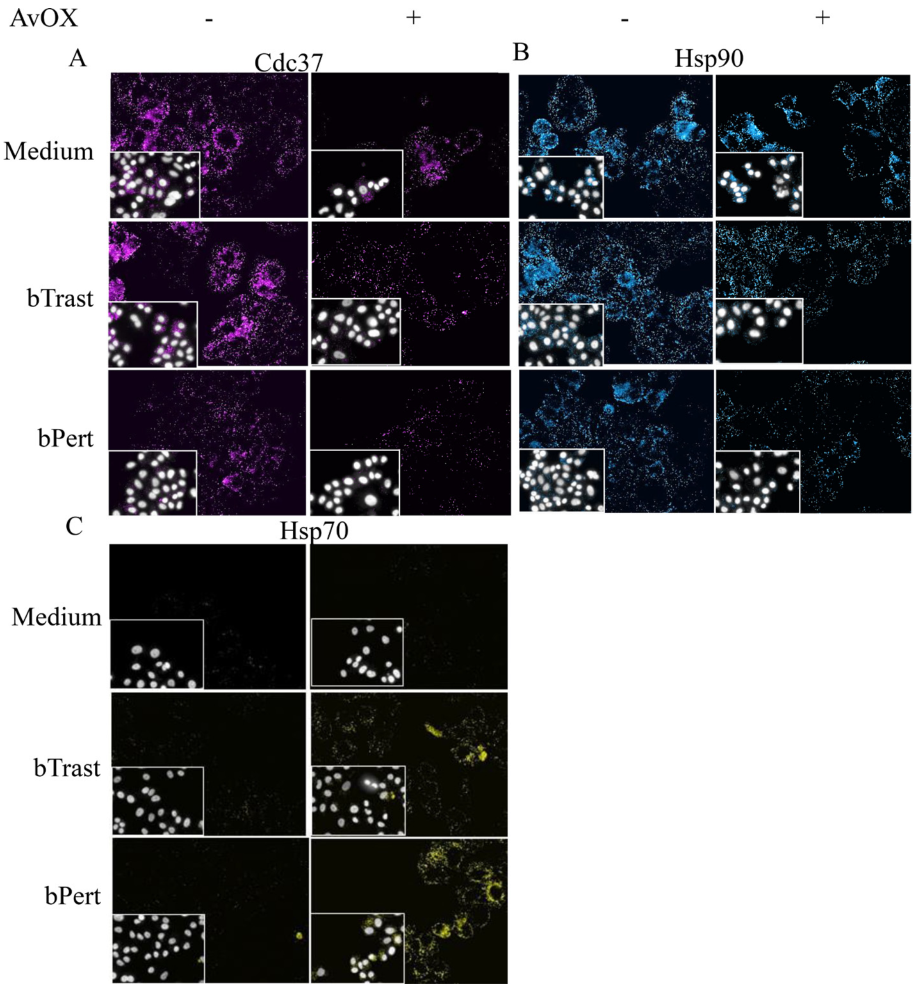

Supplementary Figure 14: AvidinOX-anchored bPert and bTrast inhibit formation of ErbB2/Cdc37-HSP90 complex and increase association of ErbB2 with Hsp70 in SKBR3 cells. Cells, with or without AvidinOX (AvOX) conjugation, were incubated 10 minutes with bTrast (5 µg/mL) or bPert (1 µg/mL). After washing, cells were cultivated 30 minutes in medium, then washed and fixed. Detection of complexes by In situ PLA. Fluorescence imaging by High Content Screening (HCS) Operetta. Each image is representative of at least 5 fields. Magnification 60x. Data are from one representative experiment out of two.
